# *Bacillus subtilis* PgcA moonlights as a phosphoglucosamine mutase in support of peptidoglycan synthesis

**DOI:** 10.1371/journal.pgen.1008434

**Published:** 2019-10-07

**Authors:** Vaidehi Patel, Katherine A. Black, Kyu Y. Rhee, John D. Helmann

**Affiliations:** 1 Department of Microbiology, Cornell University, Ithaca, NY, United States of America; 2 Division of Infectious Diseases, Weill Department of Medicine, Weill Cornell Medicine, New York, NY, United States of America; Indiana University, UNITED STATES

## Abstract

Phosphohexomutase superfamily enzymes catalyze the reversible intramolecular transfer of a phosphoryl moiety on hexose sugars. *Bacillus subtilis* phosphoglucomutase PgcA catalyzes the reversible interconversion of glucose 6-phosphate (Glc-6-P) and glucose 1-phosphate (Glc-1-P), a precursor of UDP-glucose (UDP-Glc). *B*. *subtilis* phosphoglucosamine mutase (GlmM) is a member of the same enzyme superfamily that converts glucosamine 6-phosphate (GlcN-6-P) to glucosamine 1-phosphate (GlcN-1-P), a precursor of the amino sugar moiety of peptidoglycan. Here, we present evidence that *B*. *subtilis* PgcA possesses activity as a phosphoglucosamine mutase that contributes to peptidoglycan biosynthesis. This activity was made genetically apparent by the synthetic lethality of *pgcA* with *glmR*, a positive regulator of amino sugar biosynthesis, which can be specifically suppressed by overproduction of GlmM. A gain-of-function mutation in a substrate binding loop (PgcA G47S) increases this secondary activity and suppresses a *glmR* mutant. Our results demonstrate that bacterial phosphoglucomutases may possess secondary phosphoglucosamine mutase activity, and that this dual activity may provide some level of functional redundancy for the essential peptidoglycan biosynthesis pathway.

## Author summary

Enzyme promiscuity results when an enzyme interacts with multiple, often structurally related, substrates. Phosphohexomutase family enzymes function in diverse pathways by catalyzing the isomerization of 6-phosphosugars and 1-phosphosugars. In *Bacillus subtilis*, the phosphoglucomutase (PGM) PgcA functions with glucose 6-phosphate (Glc-6-P) as substrate in support of UDP-glucose biosynthesis for glucolipid and teichoic acid synthesis. A separate phosphoglucosamine mutase (PNGM) designated GlmM functions with glucosamine 6-phosphate (GlcN-6-P) as substrate in the synthesis of aminosugars needed for peptidoglycan assembly. Here, we show that PgcA has a significant secondary activity as a PNGM and thereby contributes to PG synthesis. These results support a model in which a subset of bacterial PGM enzymes function as bifunctional PGM/PNGM enzymes, thereby providing functional redundancy for the essential process of cell wall synthesis.

## Introduction

*Bacillus subtilis* serves as a model organism for studies of cell envelope synthesis in Gram-positive bacteria. *B*. *subtilis* has a thick peptidoglycan (PG) polymer surrounding the cell that serves both as a protective barrier and to counteract internal turgor pressure. PG comprises long glycan chains with alternating subunits of N-acetyl glucosamine (GlcNAc) and N-acetyl muramic acid (MurNAc) that are crosslinked by short peptide chains [1]. The Gram-positive cell wall also contains essential anionic polymers called teichoic acids, which in *B*. *subtilis* 168 strains are alternating copolymers of glycerol and phosphate [2]. The teichoic acids are either covalently attached to the PG layer (wall teichoic acid or WTA) [3] or anchored to the membrane via a lipid (lipoteichoic acid or LTA) [4].

Bacterial cell wall synthesis and morphogenesis is strictly governed by nutrient availability. We have previously shown that the gluconeogenesis factor GlmR (previously known as YvcK) plays a critical role in diverting carbon from central carbon metabolism to PG precursor biosynthesis [5]. A *glmR* deletion mutant is unable to grow on gluconeogenic carbon sources due to a severe impairment in PG precursor biosynthesis, and this results in the sensitization of Δ*glmR* strains to various PG synthesis inhibiting antibiotics [5–7]. The essentiality of *glmR* can be bypassed by exogenously added GlcNAc, or by mutations that increase expression of the initiating enzymes of UDP-GlcNAc biosynthesis, GlmS (glucosamine 6-phosphate synthase) or GlmM (phosphoglucosamine mutase) [5–7]. These and other findings support a model in which GlmR serves to increase the activity of GlmS during gluconeogenesis, conditions where the GlmS substrate fructose-6-phosphate is present at relatively low levels [8]. Mutations affecting GlmR orthologs, such as *Mycobacterium tuberculosis* CuvA, result in similar cell morphology, antibiotic sensitivity and nutrient-dependent growth phenotypes [9], suggesting that GlmR function may be broadly conserved.

**Fig 1:**
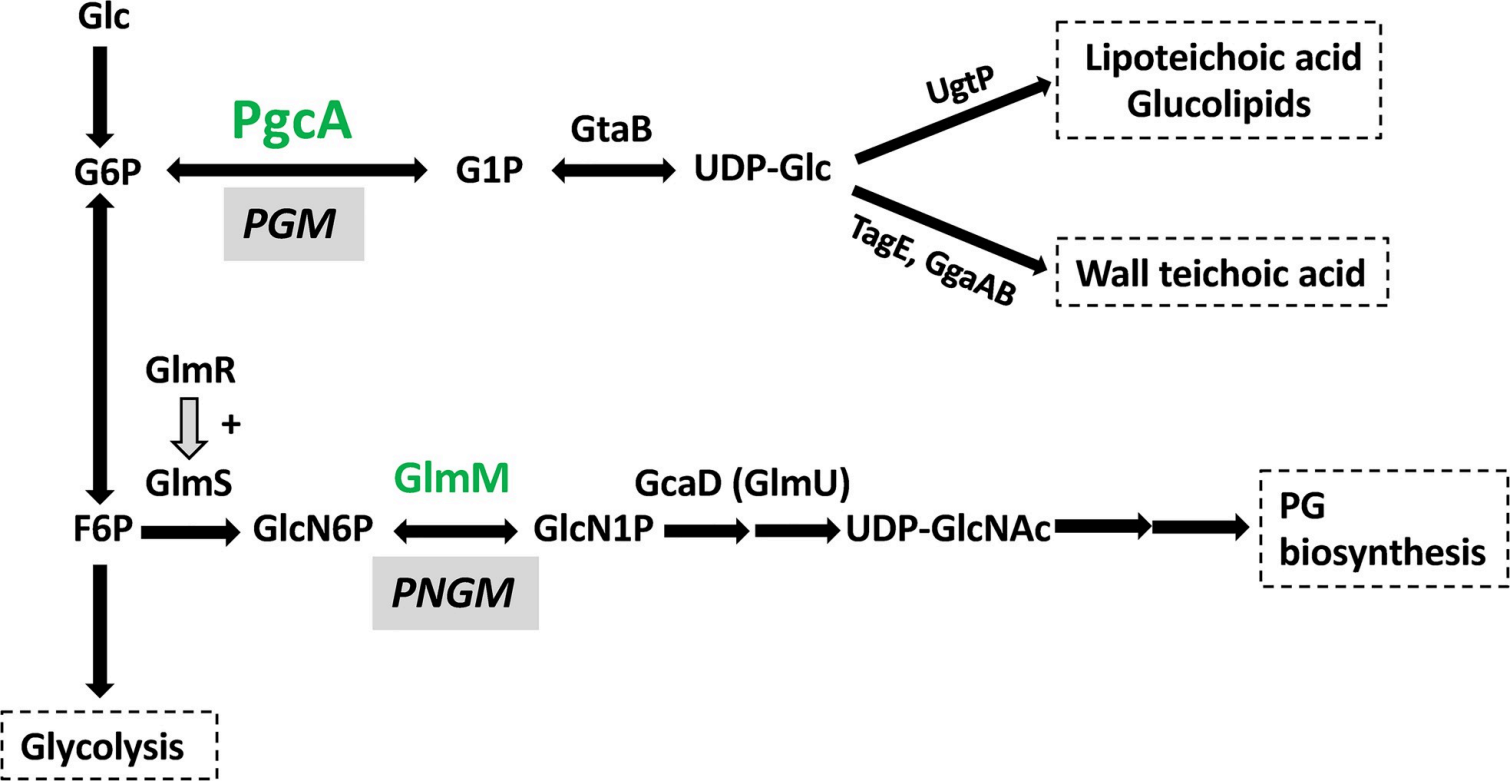
Schematic depicting the roles of PGM and PNGM enzymes in *B*. *subtilis*. Phosphoglucomutase **(**PGM) functions in glucolipid, lipoteichoic acid, and wall teichoic acid biosynthesis. In *B*. *subtilis*, PGM activity is provided by PgcA. Peptidoglycan synthesis requires phosphoglucosamine mutase (PNGM) activity. GlmM functions as a PNGM and is an essential enzyme for peptidoglycan biosynthesis.

Although a Δ*glmR* strain can grow well on LB medium, PG synthesis is impaired as judged by high sensitivity to antibiotics such as the β-lactam cefuroxime (CEF) [5]. In a previous selection for suppressors of CEF sensitivity, we recovered a point mutation in *pgcA*, encoding phosphoglucomutase, that increases CEF resistance and restores the ability of a *glmR* null mutant to grow on strictly gluconeogenic medium [5]. PgcA catalyzes the first step in UDP-glucose (UDP-Glc) biosynthesis, the conversion of Glc-6-P to Glc-1-P [10]. The second step in the pathway, catalyzed by GtaB, generates UDP-Glc from Glc-1-P and UTP (Fig 1). Next, UgtP utilizes UDP-Glc to make diglucosyldiacylglycerol (Glc_2_DAC). In *B*. *subtilis*, UDP-Glc is used for the synthesis of neutral glucolipids, which comprise ~10–15% of the lipid bilayer in exponential growth [11], the LTA membrane anchor [12], and are used for glucosylation of WTAs [3, 13, 14]. If Glc_2_DAC is not available, LTAs are synthesized on an alternative anchor, phosphatidylglycerol [15]. UgtP-dependent glucolipid synthesis also signals nutrient availability to the cell division apparatus, where UgtP antagonizes FtsZ ring assembly when UDP-Glc levels are high [16]. Thus, deletion of *pgcA*, *gtaB* or *ugtP* results in shorter cells even in nutritionally rich medium due to unavailability of UDP-Glc to serve as a metabolic signal. In addition, *ugtP* mutants have altered cell morphology and increased expression of cell envelope stress responses activated by extracytoplasmic function σ factors [17, 18].

In this study, we characterize the effect of a gain-of-function *pgcA*_G47S_ allele isolated as a suppressor of a *glmR* deletion mutant [5]. The *pgcA*_G47S_ allele functions independent of the UDP-Glc biosynthesis pathway. Genetic and biochemical studies support the notion that PgcA functions as a phosphoglucomutase (PGM, in support of UDP-Glc biosynthesis) and also moonlights as phosphoglucosamine mutase (PNGM, in support of PG synthesis). This secondary activity of PgcA is essential in a *glmR* deletion mutant: a *glmR pgcA* double mutant can only be constructed if GlmM is overexpressed to compensate for the lack of PgcA. The PgcA G47S variant increases PNGM activity several-fold. Homology modeling and protein sequence comparisons support the hypothesis that the G47-containing loop region plays a critical role in substrate selection. This hypothesis is further supported by expression and mutation studies of *E*. *coli* PgcA ortholog Pgm, which naturally has a Ser residue corresponding to *B*. *subtilis* PgcA G47, and a Pgm variant substituted with Gly. In conclusion, bacterial phosphoglucomutases may be more promiscuous than previously appreciated, and thereby serve a secondary role as phosphoglucosamine mutases that contribute to PG synthesis.

## Results

### Mutations in *glmR* and *pgcA* are synthetic lethal

We previously reported the identification of several mutations that suppressed the growth defects and antibiotic sensitivity of a *glmR* null mutant [5]. Here, we set out to better understand the mechanism of suppression by a missense mutation (*pgcA*_G47S_) in the gene encoding phosphoglucomutase. This allele suppressed both the CEF sensitivity and the inability to grow on gluconeogenic medium that is characteristic of a *glmR* null mutation [5].

**Fig 2:**
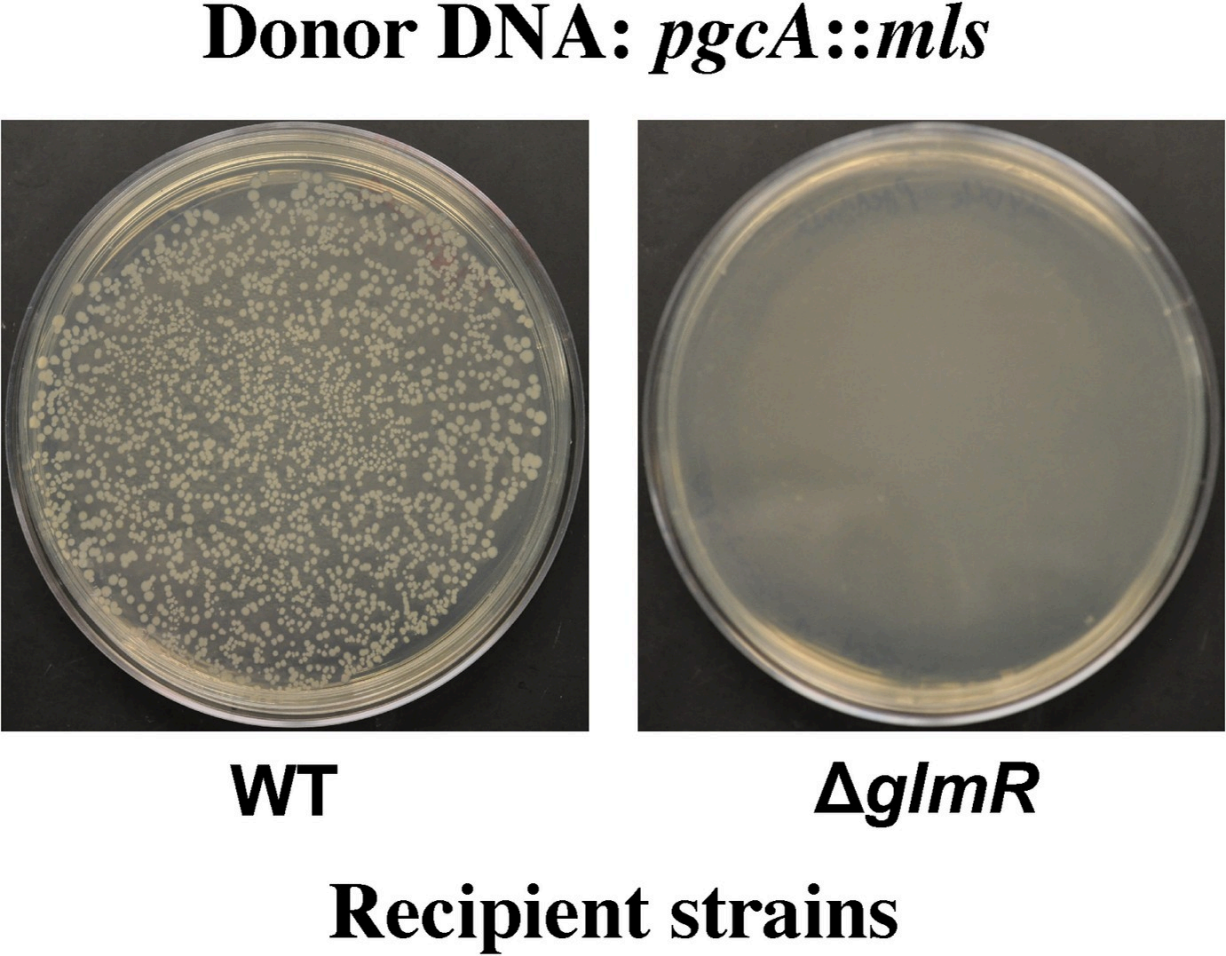
*glmR* and *pgcA* mutations are synthetic lethal. Recipient strains WT and Δ*glmR* were transformed with *pgcA*:*mls* chromosomal DNA and the transformants were selected on LB plate with MLS antibiotics and 20 mM MgSO_4_. Pictures of the transformation plates were taken after overnight incubation at 37^0^ C.

To test whether *pgcA*_G47S_ might be a loss of function or null allele, we first tried constructing a *glmR pgcA* double mutant. Recipient Δ*glmR* and wild type (WT) strains were transformed with chromosomal DNA carrying a *pgcA* allele disrupted with a macrolide-lincosamide-spectinogramin B (*mls*) antibiotic resistance cassette, *pgcA*::*mls*. Although this *pgcA* null allele can be easily transformed into WT cells, we recovered very few colonies with the Δ*glmR* strain as recipient (Fig 2, Table 1). PCR screening of these putative *glmR pgcA* transformants revealed that all of the resulting colonies arose from cells that had incorporated the *pgcA*::*mls* mutation and also received a WT copy of *glmR* by congression (cotransformation of an unlinked gene). This suggests that *pgcA* and *glmR* are synthetic lethal, at least under these selection conditions (LB medium). High magnesium (20 mM) as well as addition of glucose (1%) is known to improve the growth of *glmR* mutants [6]. Therefore, we tried constructing a *glmR pgcA* mutant with selection on plates amended with MgSO_4_ or glucose. When high magnesium was present, we did see some microcolonies, however they never increased in size after prolonged incubation, nor could they be sub-cultured. The few healthy-looking colonies obtained from the transformation plates again had a WT copy of *glmR* introduced by congression (Table 1). Thus, we were not able to construct *glmR pgcA* mutants under any of the conditions tested. In contrast, our whole-genome sequencing studies had previously identified a *glmR pgcA*_G47S_ strain that was not only viable, but grew well under gluconeogenic conditions not supportive of growth of *glmR* alone [5].

**Table 1:** Number of colony forming units (CFUs) obtained during transformation of WT and Δ*glmR* mutant strains with chromosomal DNA containing *pgcA*::*mls*.

Growth condition	WT			Δ*glmR*^1^		
	Replicate 1	Replicate 2	Replicate 3	Replicate 1	Replicate 2	Replicate 3
LB	>1200	>1330	>1500	2*	5*	8*
LB+1% Glucose	>1500	>1000	>1400	10*	3*	7*
LB+20 mM MgSO_4_	>1000	>1500	>1200	9*	1*	5*

^1^asterisks indicate that a WT copy of *glmR* was crossed back in by congression.

### *pgcA*_G47S_ is a gain of function mutation

**Fig 3:**
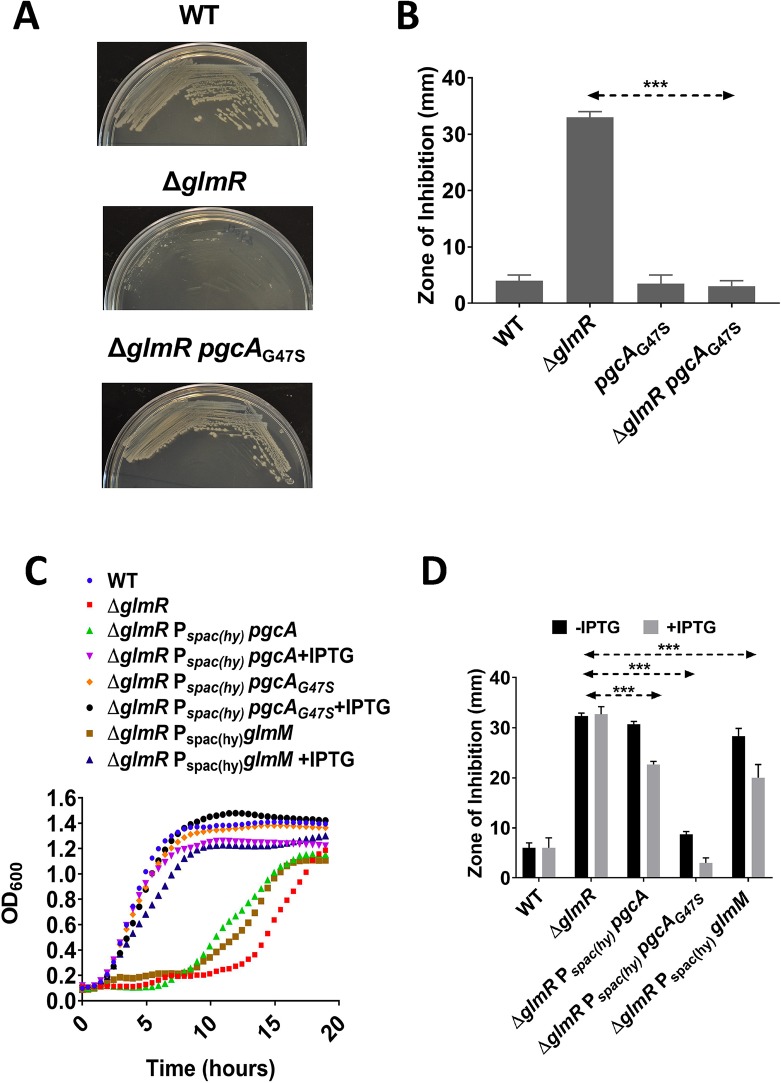
*pgcA*_G47A_ is a gain of function mutation. **(A)** Representative pictures showing growth of WT, Δ*glmR*, Δ*glmR pgcA*_G47S_ on MH and **(B)** Bar graph showing CEF sensitivity. *pgcA* and *pgcA*_G47S_ were ectopically expressed at *amyE* in Δ*glmR* and Δ*glmR* Δ*pgcA* and tested for **(C)** growth on MH medium (representative growth curves are shown; N>3) and (**D**) CEF sensitivity by disk diffusion assay using 6 μg CEF per disk. Where indicated, 1 mM IPTG was added. Standard deviation (error bars) is based on at least three biological replicates. Three asterisks represent statistical significance with P <0.001 with Tukey test.

To confirm that the *pgcA*_G47S_ allele was sufficient to restore growth of the Δ*glmR* mutant, we recreated this mutation at the native locus in a Δ*glmR* strain using CRISPR-based genome editing. As expected, this reconstructed Δ*glmR pgcA*_G47S_ strain grew on gluconeogenic Mueller Hinton (MH) medium and had reduced CEF sensitivity relative to Δ*glmR* on LB medium (Fig 3A and 3B). Thus, the *pgcA*_G47S_ allele is sufficient for *glmR* suppression.

Next, we introduced an IPTG-inducible copy of *pgcA* or *pgcA*_G47S_ into the Δ*glmR* mutant. The resulting strains were tested for growth on MH and for CEF sensitivity (Fig 3C and 3D). Induction of wild-type *pgcA* suppressed the growth defect of Δ*glmR* on gluconeogenic medium, and also led to a small reduction in CEF sensitivity. Induction of *pgcA*_G47S_ also suppressed the Δ*glmR* strain to enable growth on MH medium, and in this case strong suppression of CEF sensitivity was noted even in the absence of inducer, presumably due to leaky expression from the P_spac(hy)_ promoter. Since ectopic expression of *pgcA*_G47S_ suppresses *glmR* phenotypes even in the presence of native *pgcA*, *pgcA*_G47S_ is a dominant, gain-of-function mutation (Fig 3C and 3D). Moreover, we conclude that even increasing the level of native PgcA is sufficient for some suppression.

### The synthetic lethality of *glmR* and *pgcA* is not due to loss of UDP-glucose synthesis

**Fig 4:**
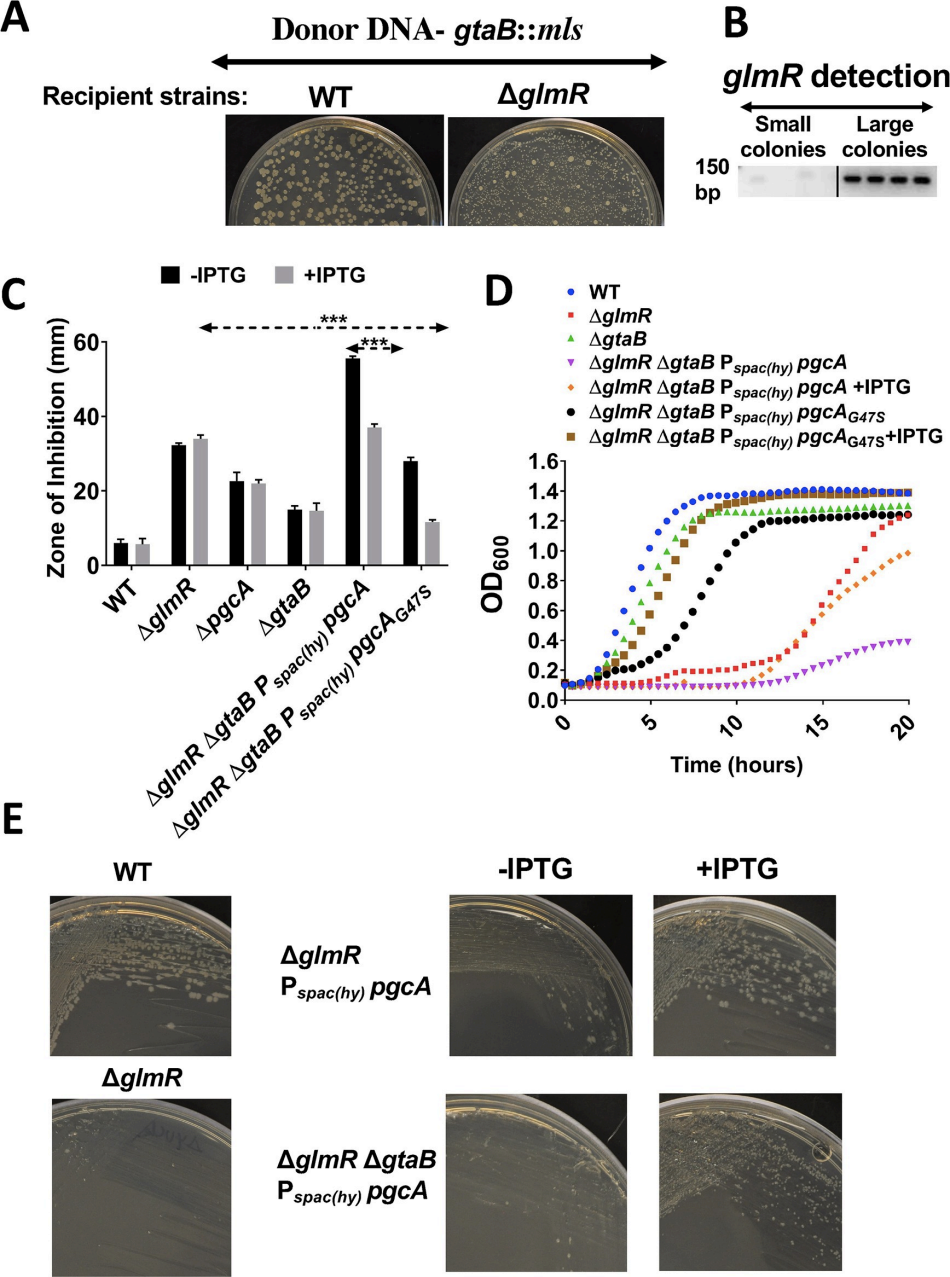
*pgcA*_G47S_ suppresses a *glmR* deletion mutation independent of UDP-Glc biosynthesis. **(A)** Representative pictures (N>3) showing LB with 20 mM MgSO_4_ plates for WT and Δ*glmR* transformation with *gtaB*::*mls* chromosomal DNA, **(B)** A representative agarose gel (N>3) electrophoresis image showing PCR amplification of Δ*glmR* with internal check primers. **(C)** CEF susceptibility assay done with 6 μg antibiotic and **(D)** representative growth curve on MH medium. Standard deviation (error bars) is based on at least three biological replicates. Three asterisks represent statistical significance with P <0.001 with Tukey test. **(E)** Growth study on MH agar plates. The pictures were taken after overnight incubation at 37^0^ C. 1 mM IPTG was added when required in all experiments.

Since PgcA is the initiating enzyme for UDP-glucose (UDP-Glc) synthesis, and we were unable to construct a *glmR pgcA* double mutant, we hypothesized that this pathway may be essential in a *glmR* null mutant strain. If this were the case, then *glmR* and *gtaB*, encoding the second enzyme in the UDP-Glc synthetic pathway, should also have a synthetic lethal relationship. However, unlike *pgcA*, we were able to successfully transform the Δ*glmR* strain with *gtaB*::*mls* chromosomal DNA. The resulting transformants, selected in the presence of 20 mM MgSO_4_, were recovered as small colonies that could be subcultured (together with larger colonies resulting from congression and reintroduction of wild-type *glmR*) (Fig 4A; S1 Fig). PCR screening confirmed that *glmR* was present in the large transformants, but not in the small colonies (Fig 4B, Table 2). Since all attempts to generate *glmR pgcA* were unsuccessful, whereas we were able to generate a *glmR gtaB* mutant, we hypothesized that PgcA has a secondary function independent of UDP-Glc biosynthesis.

**Table 2:** Transformation of *pgcA*::*mls* and *gtaB*::*mls* into recipient Δ*glmR* strain.

		Number of transformants^1^		
Recipient	Donor DNA	Replicate 1	Replicate 2	Replicate 3
Δ*glmR*	*pgcA*::*mls*	9*	3*	2*
	*gtaB*::*mls*	>600	>800	>500

^1^asterisks indicate that a WT copy of *glmR* was crossed back in by congression.

### *pgcA*_G47S_ suppresses *glmR* phenotypes independent of UDP-Glc biosynthesis

Since overproduction of PgcA suppressed the inability of a *glmR* mutant to grow on MH medium (Fig 3C), we first considered the possibility that PgcA_G47S_ simply had higher PGM catalytic activity, and that suppression resulted from an increased flux of glucose-6-phosphate to UDP-Glc (Fig 1). This possibility was ruled out by the finding that suppression of *glmR* by induction of either *pgcA* or *pgcA*_G47S_ is still observed in a strain lacking the next enzyme in the UDP-glucose biosynthesis pathway, GtaB. A *gtaB* mutant displays increased CEF sensitivity, and this is further exacerbated in a *glmR gtaB* mutant (Fig 4C). Even in this background, induction of *pgcA*, and even more dramatically *pgcA*_*G47S*_, increased CEF resistance (Fig 4C). Similarly, induction of the *pgcA*_*G47S*_ gain-of-function allele can restore the ability of the *glmR gtaB* mutant strain to grow in MH medium (Fig 4D). Growth on MH medium relies on gluconeogenesis, and this is a restrictive condition unable to support growth of *glmR* null mutant strains in the absence of a suppressor mutation [5]. Induction of the wild-type *pgcA* allele was much less effective, although growth was consistently observed after a lengthy (~11 h) lag phase (Fig 4D). A similar pattern of suppression was noted when cells were streaked on MH agar plates with and without IPTG (Fig 4E). Taken together, these observations further support the idea that PgcA has secondary role in *B*. *subtilis* apart from UDP-Glc biosynthesis, and this secondary function is enhanced by the PgcA_G47S_ mutation.

### PgcA contributes phosphoglucosamine mutase (PNGM) activity in *B*. *subtilis*

Overproduction of GlmM allows growth of a *glmR* mutant on MH medium (Fig 3C), and partially suppresses CEF sensitivity (Fig 3D), consistent with prior results [5]. PgcA and GlmM are both enzymes from the phosphohexomutase (PHM) superfamily, and they have the same catalytic mechanism [19]. PgcA has PGM activity and recognizes glucose 6-P or glucose 1-P (with a 2-hydroxyl group), whereas GlmM has PNGM activity and recognizes glucosamine 6-P or glucosamine 1-P (with a 2-amino group) (Fig 1). Previous studies provide precedence for the idea that PHM family enzymes may be promiscuous and act on multiple substrates [20–22]. We therefore hypothesized that PgcA has significant PNGM activity, and that this activity may account for the synthetic lethality of *glmR* and *pgcA*. Moreover, we suggest that G47S increases this secondary activity.

**Fig 5:**
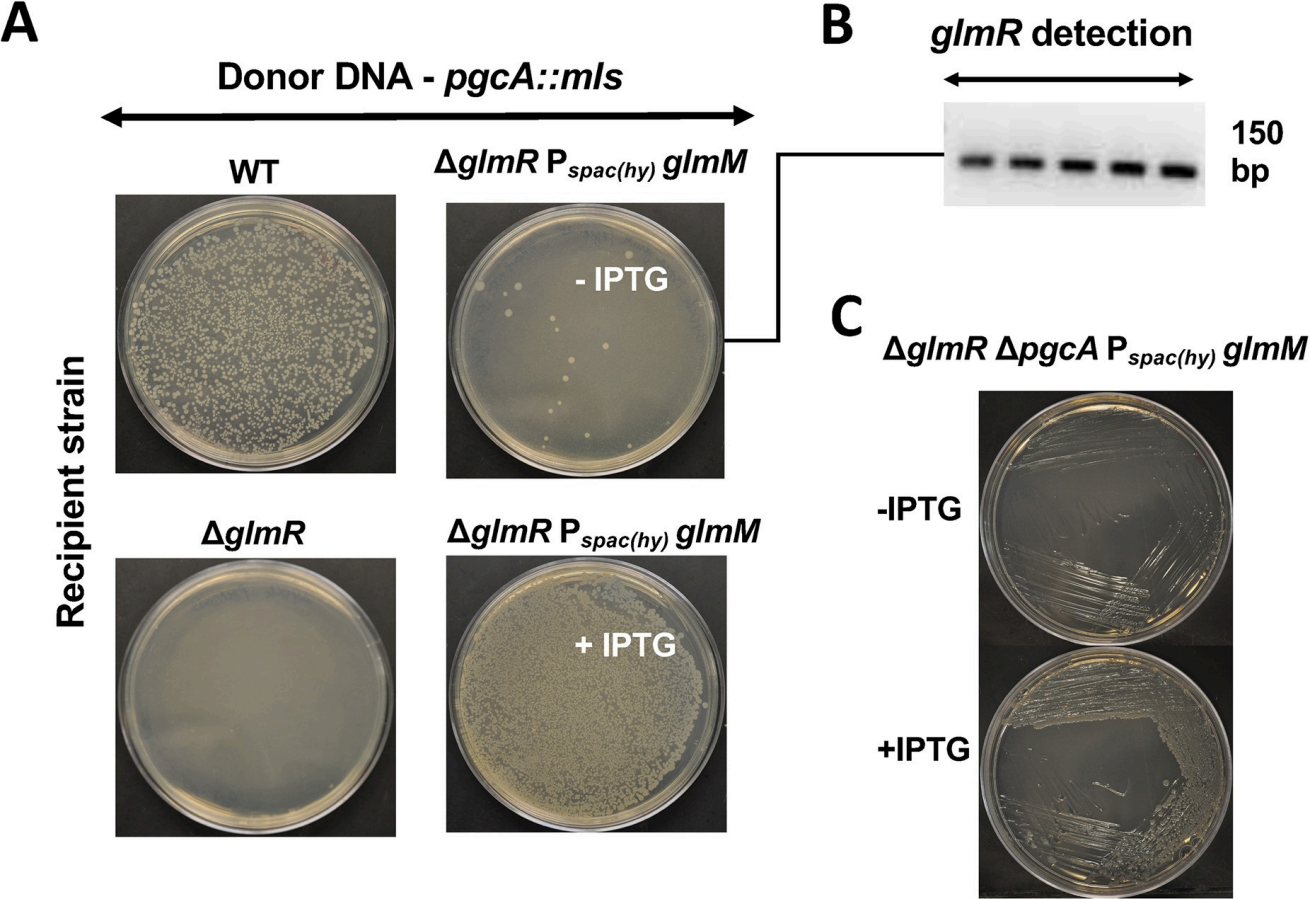
PgcA has PNGM activity *B*. *subtilis*. **(A)** WT, Δ*glmR and* Δ*glmR P*_*spac(hy)*_ *glmM* transformation carried out with *pgcA*::*mls* strain chromosomal DNA. Transformants were selected on LB containing MLS antibiotics and 20 mM MgSO_4._ 1mM IPTG was added to the medium when necessary. **(B)** A representative agarose gel (N>3) electrophoresis image showing PCR amplification of Δ*glmR* with internal check primers. **(C)** Representative pictures showing growth phenotype of Δ*glmR* Δ*pgcA* P_spac(hy)_*glmM* on MH agar plate with and without 1 mM IPTG.

To test the idea that *pgcA* is essential in the *glmR* background because it is necessary to provide sufficient PNGM activity to support growth, we tried deleting *pgcA* from a *glmR* deletion strain with an inducible copy of *glmM*. Transformants were selected on LB medium containing 20 mM Mg^2+^ to support growth of cells with compromised cell wall function. Healthy Δ*glmR* Δ*pgcA amyE*::P_*spac(hy)*_ *glmM* transformants were only obtained at high frequency on plates containing 1mM IPTG, and therefore overexpressing GlmM (Fig 5A). These transformants were viable only when *glmM* was induced with IPTG (Fig 5C). The few colonies that grew in absence of IPTG had a WT copy of *glmR* crossed back into the strain, as confirmed with PCR screening (Fig 5B). We also tried deleting *pgcA* from *glmR* strains overexpressing either *glmS* or *gcaD* (*glmU)*. Overexpression of either GlmS, GlmM or GcaD can suppress the inability of Δ*glmR* to grow on MH medium [5], but only GlmM can bypass the synthetic lethality upon deletion of *pgcA* (Table 3). We conclude that *pgcA* is dispensable in the *glmR* strain only if GlmM is overproduced, and overproduction of enzymes that catalyze reactions before or after GlmM (Fig 1) does not compensate for loss of *pgcA*. The converse experiment was not successful, we were unable to delete *glmM* from strains overexpressing either PgcA or the PgcA_G47S_ variant, suggesting that either the level of PNGM activity from these proteins is insufficient to support growth, or that GlmM has other essential functions that cannot be replaced by PgcA.

**Table 3:** Transformation of *pgcA*::*mls* into recipient strains expressing PG biosynthetic enzymes induced with 1 mM IPTG^1^.

Recipient Strains (+IPTG)	Replicate 1	Replicate 2	Replicate 3
Δ*glmR* P_spac(hy)_*glmM*	>1000	>1400	>1500
Δ*glmR* P_spac(hy)_*glmS*	5*	2*	7*
Δ*glmR* P_spac(hy)_*gcaD* (*glmU)*	3*	4*	8*

^1^asterisks indicate that a WT copy of *glmR* was crossed back in by congression

### PgcA has PGM and PNGM activity, and PgcA G47S selectively increases PNGM activity

To test the notion that PgcA has intrinsic PNGM activity that is increased in the PgcA G47S variant we purified both proteins and assayed activity *in vitro*. Native PgcA and the PgcA G47S variant were incubated in buffer with 20 μM glucose-1,6-bisphosphate, which facilitates formation of the active phosphorylated form of the enzyme [23], and Glc-1-P was used as substrate to assay the rate of substrate isomerization. Both PgcA and PgcA G47S were equally active in the PGM reaction, with indistinguishable kinetic parameters (S2 and S3A Figs).

When the reactions were repeated using GlcN-1-P as substrate to monitor the PNGM reaction, the observed catalytic rate (k_cat_) for PgcA G47S was nearly 4-fold higher than for PgcA, with little change in K_M_ (S3B Fig). Correspondingly, the PgcA G47S protein had a >4-fold increase in catalytic efficiency (k_cat_/K_M_) in the PNGM reaction relative to the PgcA protein (40.14 vs. 9.38 mM^-1^ s^-1^, respectively) (S3B Fig). The specific activities measured here for PgcA in the PNGM reaction using 1.5 mM GlcN-1-P as substrate (3 μmol min^-1^ mg^-1^ for WT, ~10 μmol min^-1^ mg^-1^ for PgcA G47S; S1 Table) are comparable to the highest reported specific activities for GlmM proteins, including that purified from *E*. *coli* and assayed after activation with GlcN-1,6-bisphosphate [21].

We also purified GlmM from *B*. *subtilis*, which had an affinity for GlcN-1-P substrate comparable to that measured for PgcA with Glc-1-P, as judged from the K_M_, but displayed overall much lower catalytic activity (S2B and S3B Figs, S2 Table). This may indicate that the *in vitro* activation of the GlmM enzyme to the phosphorylated form by glucose-1,6-bisphosphate, which has been routinely included as an activating compound for most studied GlmM proteins [23], was inefficient under our conditions. We also tested ATP and fructose-1,6-bisphosphate as possible phosphodonors to activate the purified PgcA and GlmM proteins, but without any stimulation of activity noted. It is possible that *in vivo* there is a specific kinase involved, or perhaps some other physiological phosphodonor, but this is currently unresolved [21, 23].

### G47 is located in an active site loop region

**Fig 6:**
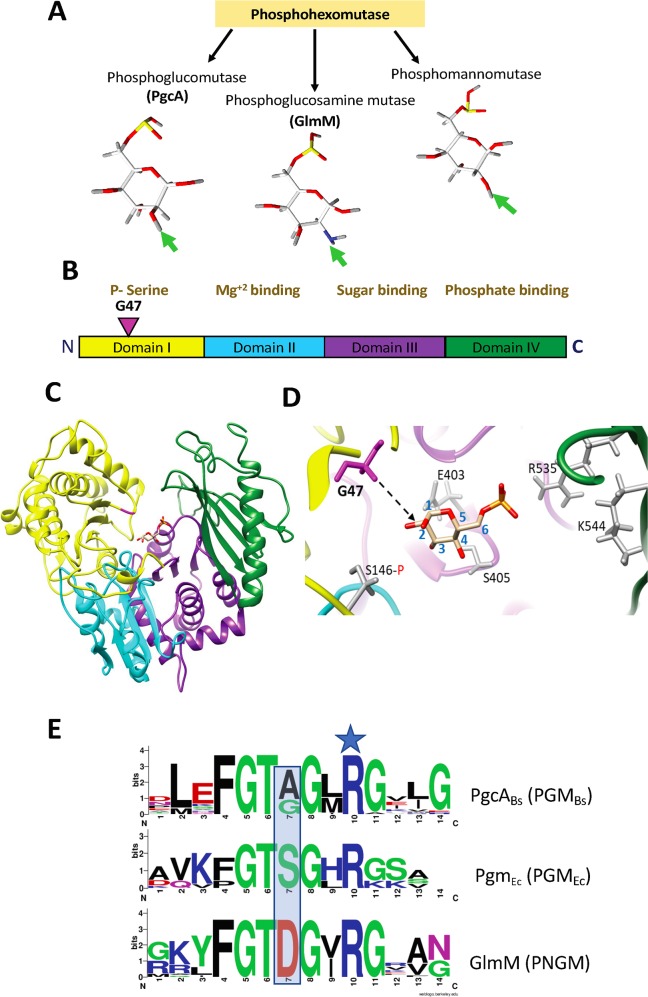
Identification of a mutation affecting substrate-selectivity in PGM/PNGM family enzymes. **(A)** Schematic depicting enzymes from the phosphohexomutase family and their 6-phosphohexose sugar substrates, with the variable substituents at the C2 position highlighted by an arrow. (**B**) domain structure and conserved functions within PGM enzymes, as detailed in [22]. N and C indicate the N-terminus and C-terminus of the enzyme, respectively. The pink inverted triangle indicates residue G47 in domain I. **(C)** *B*. *subtilis* PGM (PgcA) modelled with I-TASSER using *P*. *aeruginosa* bifunctional PGM/PMM (PDB#1P5G; which contains bound glucose 6-phosphate) as a scaffold (25% identical to *B*. *subtilis* PgcA) illustrating the position of the bound glucose 6-phosphate in the substrate-binding cleft. **(D)** Enlarged view of the substrate-binding site of modeled *B*. *subtilis* PgcA. Residue G47 is illustrated in pink with a black broken arrow indicating the relationship of the protein backbone in this region to the carbon-2 position of bound glucose 6-phosphate. The grey residues include functionally important residues of the active site including the serine (S146) that is involved in phosphoryl transfer (also located in domain I), as well as previously described sugar phosphate binding residues (E403, S405, R535, K544; *B*. *subtilis* numbering) of domain IV. As per the homology modelling, E403 and S405 would interact with the sugar backbone, whereas R535 and K544 form salt bridges with the bound glucose 6-phosphate. (**E**) Sequence Logo representation of conserved amino acids in the loop region adjacent to an invariant active site Arg residue (blue star) in three groups of PGM/PNGM enzymes including 24 bacterial PGM enzymes related to *B*. *subtilis* PgcA (designated as the PGM_Bs_ group) and defined by a conserved motif with an Ala/Gly at the position corresponding to PgcA Gly47 (residue 7 in the top motif), 8 proteobacterial PGM enzymes including *E*. *coli* Pgm (designated as the PGM_Ec_ group) defined by a Ser residue at this position (middle motif), and 20 bacterial PNGM (GlmM) enzymes with an Asp at this position. Sequence Logos were derived from the aligned sequences in S4 and S5 Figs.

PHM family enzymes function on a variety of related sugars that differ in the nature and orientation of the substituent on position 2 of the hexose (Fig 6A). To better understand the effect of the G47S substitution, we used I-TASSER to model *B*. *subtilis* PgcA on the structure of the well characterized *Pseudomonas aeruginosa* bifunctional phosphoglucomutase/phosphomannomutase (PGM/PMM) (PDB#1P5G). *P*. *aeruginosa* PGM/PMM is homologous to *B*. *subtilis* PgcA (25% identity) and GlmM (28% identity), with a very conserved catalytic site. The PHM family enzymes are very similar in their overall tertiary structure [17], with four domains surrounding a large catalytic cleft (Fig 6C). Each domain possesses residues for substrate recognition and/or catalysis (Fig 6B and 6C). The PgcA G47 residue aligns with Y17 of *P*. *aeruginosa* PGM/PMM and maps to a loop region in domain I (Fig 6B), which forms part of the substrate-binding site (Fig 6C and 6D). This loop region is proximal to the sugar 2 position that distinguishes glucose from glucosamine substrates in structures of enzymes bound to 6-phosphosugars [24].

To further explore the notion that this region may play a role in substrate selection, we generated multiple-sequence alignments for PHM enzymes from different families. Alignment of PGM from various bacteria revealed that Gly and Ala are found at the position of PgcA G47 in *Firmicutes*, some actinobacteria and some proteobacteria (S4 Fig). However, in some proteobacteria (including *Escherichia coli*) Ser is found at this position (S4 Fig). In contrast, alignment of PNGM enzymes (e.g. GlmM) reveals that the corresponding residue is typically Asp (S5 Fig). To determine if these changes corresponding to PgcA position 47 are correlated with other changes in this region, these multisequence alignments were used to generate SequenceLogo motifs corresponding to each of these three groups of enzymes. Indeed, the proteins mostly closely related to *B*. *subtilis* PgcA in this loop region (PGM_Bs_), to *E*. *coli* Pgm (PGM_Ec_), and to GlmM differ in their pattern of conservation (Fig 6E). The corresponding position in a bifunctional *P*. *aeruginosa* PGM/PMM is Tyr17, previously proposed based on phylogenetic comparisons to be a class specific residue in this family of enzymes [19]. Our results further support the proposal that PgcA G47 corresponds to a class-specific residue in the PHM superfamily.

In addition to this class-specific residue, this loop region also contains conserved residues that are important in catalysis. Specifically, the highly conserved Arg residue in this loop is positioned proximal to the site of phosphoryl transfer (with phosphate carried by an active site Ser), and is proposed to function as a general base to abstract a proton from the hydroxyl group of the recipient hydroxyl during phosphoryl transfer [25]. The conserved Thr residue immediately adjacent to G47 is proposed to function as part of a hydrogen-bonded latch that connects conserved domains I and IV to help generate the closed conformation of the enzyme [26].

In the PNGM (GlmM) sub-family enzymes, the residue corresponding to G47 in *B*. *subtilis* PgcA is often an Asp (S5 Fig). Since the PgcA G47S substitution increased intrinsic PNGM activity, we speculated that G47D substitution might increase this activity even further. To test this, we created a *pgcA*_G47D_ mutation at the native locus with CRISPR-editing and tried introducing a *glmR* deletion mutation in the strain. However, we were not able to create a *glmR pgcA*_G47D_ double mutant (S6 Fig). This suggests that rather than having increased PNGM activity, this allele may encode a non-functional protein. We did get a few colonies, but in each case a WT copy of *pgcA* was crossed back into these isolates. As expected, we were easily able to transform *glmR*::*mls* into both WT and *pgcA*_G47S_ recipients (S6A Fig). Additionally, *pgcA*_G47D_ is as sensitive to CEF as Δ*pgcA*, and overexpression of *pgcA*_G47D_ does not reduce CEF sensitivity of Δ*glmR* (S6B Fig). Similar to a *pgcA* null mutant, the *pgcA*_G47D_ strain does not plate SPP1 phage (S6C Fig). These data suggest that the PgcA G47D mutation behaves genetically as a null mutation, and that this change inactivates PgcA. In support of this notion, purified PgcA G47D protein was reduced 99% in the PGM assay and 90% in the PNGM assay relative to the WT values reported above (S2 and S3 Figs).

### *Escherichia coli* phosphoglucomutase (Pgm) overexpression suppresses Δ*glmR*

**Fig 7:**
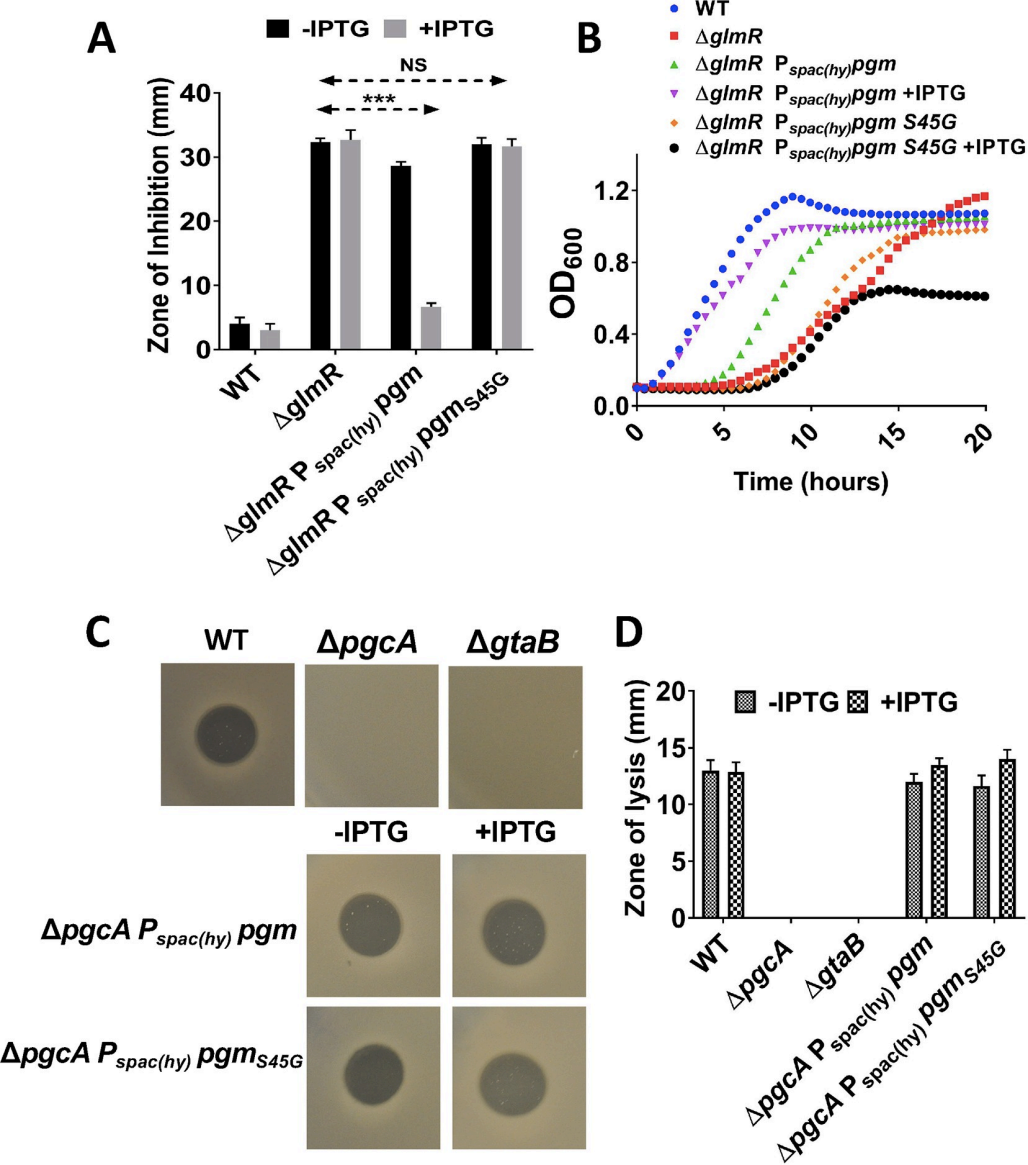
*Escherichia coli* phosphoglucomutase (Pgm) overexpression suppresses Δ*glmR* phenotypes in *B*. *subtilis*. **(A)** Bar graph showing CEF sensitivity assay. Three asterisks represent statistical significance with P <0.001 with Tukey test. **(B)** Representative growth curve (N>3) study in MH medium, **(C)** Images and **(D)** Bar graph showing SPP1 infection mediated zone of lysis. 10 μl of 10^7^ PFU/ml SPP1 was spotted on the lawns *B*. *subtilis* strains of interest. Cell lysis (plaque formation) was observed after overnight incubation at 37^0^ C. Images are representative of at least three biological replicates, and error bars represent standard deviation.

In some proteobacteria, including *E*. *coli*, Ser is found at the position corresponding to PgcA G47 in the corresponding PGM (Pgm) (S4 Fig). Since our results with *B*. *subtilis* PgcA suggest that a Gly to Ser change increases the associated PNGM activity, we wished to test whether the *E*. *coli* ortholog (Pgm) could also contribute PNGM activity. Indeed, a significant increase in CEF resistance was observed when *E*. *coli* Pgm was induced in a *B*. *subtilis* Δ*glmR* strain (Fig 7A) and growth on MH medium was also restored (Fig 7B, S7 Fig). These results suggest that *E*. *coli* Pgm is also a bifunctional PGM/PNGM when expressed in *B*. *subtilis*.

Next we generated a Pgm S45G variant, and induction of this *pgm*_S45G_ allele did not reduce CEF sensitivity or suppress the growth impairment of Δ*glmR* on gluconeogenic MH medium (Figs 7B and S3). Thus, Pgm carrying an S45G substitution seems to lack sufficient PNGM activity for suppression of the *glmR* defect. To determine if the Pgm S45G variant had retained PGM activity, we used an SPP1 phage infection assay. UDP-Glc produced from the catalytic activities of PgcA and GtaB is utilized for glucosylation of WTA [3, 10]. Glucosylated WTA serves as the receptor for certain bacteriophage and facilitates their entry into *B*. *subtilis* [27–29]. In an SPP1 infection study, we observe that Δ*pgcA* and Δ*gtaB* are resistant to the infection, consistent with previous observations [28] (Fig 7C and 7D). Ectopic expression of *E*. *coli* Pgm complements the *pgcA* deletion mutant and we observe plaque formation due to SPP1 absorption and cell lysis (Fig 7C and 7D). When Pgm S45G was overexpressed in Δ*pgcA*, we still observed plaque formation, suggesting that this enzyme has retained PGM activity (Fig 7C and 7D).

## Discussion

The growth of cells is critically dependent on the ability to properly coordinate the flux of metabolites by the dynamic regulation of enzyme activity. We previously described GlmR (formerly YvcK) as a regulator of the branchpoint between central carbon metabolism and peptidoglycan synthesis [5]. The GlmR protein interacts with GlmS, which functions to divert the glycolysis/gluconeogenesis intermediate fructose-6-phosphate to glucosamine-6-phosphate (GlcN-6-P), an intermediate needed to generate the aminosugars used in peptidoglycan biosynthesis. Cells lacking *glmR* have a number of distinct phenotypes, including aberrant cell morphology [6, 7], sensitivity to PG synthesis inhibitors [30], and an inability to grow using gluconeogenesis [5, 6], a condition that leads to a significantly reduced pool of fructose-6-phosphate relative to growth on glucose [8].

Most mutations that suppressed these *glmR* phenotypes led to an increase in expression of either GlmS or the immediate downstream enzyme GlmM [5]. For example, a *glmS1* ribozyme mutation prevents the feedback inhibition of GlmS synthesis by its product (GlcN-6-P), and elevated GlmM protein likely reduces this feedback inhibition by conversion of GlcN-6-P to the next intermediate, GlcN-1-P. Consistent with a key role for GlmR in activating the GlmS reaction, *glmR* can be chemically complemented by feeding cells with N-acetylglucosamine, which feeds into metabolism as GlcN-6-P, and this chemical complementation is most robust when GlcN-6-P cannot be redirected to central metabolism due to mutation of the *nagA* and *gamB* genes. Our results support a model in which GlmR is critical for the proper diversion of carbon into PG synthesis, and the ability of UDP-GlcNAc to bind GlmS likely serves to counter this stimulatory activity [5, 7]. We also reported that the *glmR* mutation could be suppressed by a missense mutation in *pgcA* [5], and we here explore the mechanism of suppression mediated by this *pgcA*_G47S_ mutation.

PgcA is a member of the large phosphohexomutase (PHM) superfamily of enzymes that catalyze the reversible intramolecular phosphoryl transfer reaction on sugar substrates [22]. The PHM superfamily includes phosphoglucomutase (PGM), phosphomannose mutase (PMM), phosphoglucosamine mutase (PNGM), and the eukaryote-specific enzyme phospho-N-acetylglucosamine mutase (PAGM). PgcA is classically considered to be a PGM and is required in *B*. *subtilis* for the synthesis of neutral glucolipids and functions in pathways for teichoic acid synthesis and modification. We were struck by the fact that PgcA and GlmM are both members of the PHM superfamily, and were led to the hypothesis that PgcA might be a bifunctional enzyme with both PGM and PNGM activities.

PHM enzymes are generally assumed to function with high selectivity *in vivo*, although some exceptions have been noted [22]. For example, some organisms have bifunctional PMM/PGM enzymes. Amino acid residues within the substrate binding site determine substrate selectivity, and sequence comparisons have identified class-specific residues implicated in substrate selection [19]. In the specific case of a bifunctional *P*. *aeruginosa* PMM/PGM, these class-specific residues included a Tyr residue at the position explored here. Our results further support the assignment of this position as a class-specific position. Sequence alignments reveal that this position is correlated with selectivity, with PGM enzymes having Gly/Ala/Ser and PNGM enzymes often having Asp (Fig 6E). In *Trypanosoma brucei*, a dedicated PGM is absent and is substituted by the promiscuous activity of two related PHM family enzymes, PMM and PAGM [20]. Interestingly, PAGM from *T*. *brucei* has a Ser at this position (S8 Fig). Previously, it was observed that *E*. *coli* PNGM (GlmM) displays a low level of PGM activity (1400-fold lower than PNGM), and this secondary activity was enhanced 20-fold by a single S100T mutation near the substrate binding site [21, 31].

Here we show that *B*. *subtilis* PGM (PgcA) possesses PNGM activity (~20-fold less than PGM activity) and can thereby contribute, together with GlmM, to the synthesis of aminosugars needed for peptidoglycan (PG) biosynthesis. Thus, at least some PGM enzymes may be considered as bifunctional PGM/PNGM enzymes. Support for the notion that this secondary PNGM activity is physiologically relevant comes from the synthetic lethality of null mutations affecting *B*. *subtilis* PGM (*pgcA*) and GlmR, an activator that stimulates GlmS function, which provides the substrate for the major PNGM, GlmM. Null mutations in *glmR* are restricted for carbon flow into PG synthesis and are known to be suppressed by increased GlmM activity [5]. Indeed, only when GlmM is overproduced is it possible to construct a *pgcA glmR* double mutant. This suggests that PgcA functions, even in wild-type cells, as a bifunctional PGM/PNGM and contributes significantly to PG biosynthesis. Similarly, our genetic results suggest that *E*. *coli* PGM, which naturally has a Ser residue at this position, can provide PNGM activity when expressed in *B*. *subtilis*. However, this PNGM activity is no longer physiologically relevant in the converse variant, in which this Ser is replaced by Gly, although complementation studies suggest that this protein retains PGM activity (Fig 7).

Although PgcA G47S has increased PNGM (GlmM) activity, and this bypasses the essentiality of *glmR* on gluconeogenic carbon sources, this PNGM activity does not allow PgcA to substitute for the essential role(s) of GlmM. Similarly, *E*. *coli* PGM overexpression did not allow growth of a thermosensitive *E*. *coli glmM* mutant at the restrictive temperature [31]. We favor the notion that GlmM may have a second essential function. In PG biosynthesis there are numerous examples of multicomponent protein complexes [32]. In *B*. *subtilis*, the cytoplasmic protein GlmM localizes at the membrane using c-di-AMP synthase CdaA as an anchor protein [33], and may additionally interact with other proteins important for PG biosynthesis. Cyclic-di-AMP is an essential signaling nucleotide that modulates cellular osmolarity, and thus maintains cell envelope integrity by controlling potassium homeostasis [34]. GlmM controls CdaA enzymatic activity by protein-protein interaction [33, 35, 36]. Therefore, deletion of GlmM may disrupt c-di-AMP signaling and thereby affect cell viability. The ability of *E*. *coli* GlmM to substitute for *B*. *subtilis* GlmM suggests that this interface may be conserved [37], or that the critical function of GlmM involves interactions with other proteins.

In conclusion, our genetic analysis show that the *B*. *subtilis* PGM PgcA has PNGM activity which can be further enhanced by a G47S substitution. Our study also highlights that PHM class specific residue G47 in this active site loop is important for substrate recognition: glycine correlates with PGM activity, and serine with increased PNGM activity. However, even native PgcA (with G47) has PNGM activity (~20-fold lower than PGM activity), and our results demonstrate that this functions in support of PG synthesis. However, this PNGM activity does not allow PgcA to fully substitute for all GlmM functions since the *glmM* gene is still essential, and cannot be functionally replaced by non-orthologous enzymes despite their substantial PNGM activity.

## Materials and methods

### Bacterial strains and growth conditions

*B*. *subtilis* strains used are derived from strain 168 (*trpC2*) and the strains are listed in Table 4. Primers used in strain construction are in S3 Table. *E*. *coli* strain DH5α was used for cloning. Bacteria were cultured in LB broth for all the assays described later at 37^0^ C with vigorous shaking. *B*. *subtilis* strains with *glmR* mutation were streaked on LB agar plate with 20 mM MgSO_4_ and the selection antibiotic. Antibiotics were added to growth media when required at the following concentrations: 100 μg ml^-1^ ampicillin, 30 μg ml^-1^ chloramphenicol for *E*. *coli*, 10 μg ml^-1^ kanamycin, 10 μg ml^-1^ chloramphenicol, 5 μg ml^-1^ tetracycline, 100 μg ml^-1^ spectinomycin and 1 μg ml^-1^ erythromycin with 25 μg ml^-1^ lincomycin (*erm*; macrolide-lincomycin-streptogramin B resistance).

**Table 4:** *B*. *subtilis* strains used in this study.

Strain	Genotype	Reference/construction^1^
168	*trpC2*	Lab stock
HB16820	*trpC2 pgcA*::*mls*	ChrDNA of BKE *pgcA*::*mls→* lab WT 168
HB16841	*trpC2 gtaB*::*mls*	ChrDNA BKE *gtaB*::*mls→* lab WT 168
HB16848	*trpC2 glmR*	[5]
HB16910	*trpC2 glmR amyE*::P_spac(hy)_ *glmM*	Patel *et al*. (2018)
HB16945	*trpC2 glmR amyE*::*P*_*spac(hy)*_ *pgcA*	pPL82-*pgcA→* HB16848
HB16946	*trpC2 glmR amyE*::*P*_*spac(hy)*_ *pgcA*_*G47S*_	pPL82-*pgcA*_*G47S*_*→* HB16848
HB21922	*trpC2 glmR amyE*::P_spac(hy)_ *glmU*	[5]
HB21942	*trpC glmR amyE*::P_spac(hy)_ *glmS*	[5]
HB21981	*trpC2 glmR gtaB*::*mls amyE*::*P*_*spac(hy)*_ *pgcA*	ChrDNA of HB16841*→* HB16945
HB21983	*trpC2 glmR gtaB*::*mls amyE*::*P*_*spac(hy)*_ *pgcA*_*G47S*_	ChrDNA of HB16841*→* HB16946
HB21986	*trpC2 glmR pgcA*::*mls amyE*::*P*_*spac(hy)*_ *glmM*	ChrDNA of HB16820*→* HB16910
HB22014	*trpC2 pgcA*_*G47S*_	CRISPR editing at native locus in WT168
HB22020	*trpC2 glmR pgcA*_*G47S*_	CRISPR editing at native locus in HB16848
HB22021	*trpC2 pgcA*_*G47D*_	CRISPR editing at native locus in WT168
HB22025	*trpC2 pgcA*_*G47S*_ *amyE*::*P*_*spac(hy)*_ *glmM*	ChrDNA of HB16910*→* HB22014
HB22026	*trpC2 pgcA*_*G47D*_ *amyE*::*P*_*spac(hy)*_ *glmM*	ChrDNA of HB16910*→* HB22021
HB22027	*trpC2 glmR amyE*::*P*_*spac(hy)*_ *pgcA*_*G47D*_	pPL82- *pgcA*_*G47D*_*→* HB16848
HB22028	*trpC2 glmR amyE*::*P*_*spac(hy)*_ *E*. *coli pgm*	pPL82-*pgm→* HB16848
HB22042	*trpC2 glmR amyE*::*P*_*spac(hy)*_ *E*. *coli pgm*_S45G_	pPL82-*pgm*_S45G_*→* HB16848

^1^ The donor DNA and recipient strain are shown before and after the arrow respectively.

### Cloning, transformation and strain construction

For cloning procedures, restriction digestion and T4 ligation was carried out as per NEB protocols. *E*. *coli* DH5α was used for cloned vector transformation and amplification. Cloning was confirmed by polymerase chain reaction (PCR) followed by Sanger’s sequencing. *B*. *subtilis* transformation was carried out in minimal competence media with 10 mM K_2_PO_4_ pH 7.0, 3 mM sodium citrate, 10 mM of MgSO_4,_ 0.1 mM ferric ammonium citrate, 0.5 mM tryptophan, 0.2% sodium glutamate and 0.1% casein hydrolysate. DNA was added when the OD_600_ of the recipient strain reached ~0.7–0.8. After one hour of incubation at 37^0^ C, cells were plated on LB agar plate with the necessary antibiotic for selection. If the strain had a *glmR* mutation, 20 mM MgSO_4_ was added to the transformant selection LB agar plates unless specified otherwise. IPTG was added to the final concentration of 1 mM in transformation medium and transformant selection LB agar plate when required. Internal check primer pair, (Forward) CCGTCTGCGGAATGAACTGAAA and (Reverse) TTCCCGAGAGAATGGCCTGTTA were used for *glmR* deletion mutant screening. Vector pPL82 was used for all the IPTG inducible gene overexpression constructs at *amyE* locus [38]. CRISPR mediated mutagenesis was carried out as described previously [5, 39]. All *B*. *subtilis* bacillus knock out erythromycin (BKE) deletion mutants were obtained from the *Bacillus* genomic stock center (BGSC). The *erm(mls)* cassette from these strains was removed using pDR244 [40] as described previously [5].

### Antibiotic susceptibility assay

Antibiotic sensitivity was tested using disk diffusion assays, which were carried out on LB medium. Strains to be tested were grown in 5 ml LB broth at 37^0^ C with vigorous shaking to an OD_600_ of ~0.4. 100 μl of cells were added to 4 ml top LB agar (0.7% agar) kept at 48^0^ C. 1 mM IPTG was added to top agar when indicated. Top agar with cells was poured over 15 ml LB bottom agar (1.5%) plate. A Whatman paper disk (7 mm diameter) with 6 μg CEF was put on the plate unless specified otherwise. Plates were incubated at 37^0^ C overnight and the clear zone of inhibition was measured the next day.

### Growth assay on MH

To test the ability of *B*. *subtilis* mutants to grow on gluconeogenic medium we used MH medium (Sigma-Aldrich, USA) prepared per the manufacturer's instruction. Growth was monitored using a Bioscreen growth analyzer with 200 μl of MH broth in 100 well Bioscreen plates inoculated with 2 μl of *B*. *subtilis* strains pre-grown in LB broth at 37^0^ C to an OD_600_ of ~0.4. When required, glucose, MgSO_4_ and IPTG was added to the final concentrations of 1%, 20 mM and 1 mM respectively.

### Phage infection analysis

The strains of interest were grown in TY broth (LB medium supplemented with 10 mM MgSO_4_ and 10 μM MnSO_4_) at 37^0^ C to OD_600_ of ~0.8. 200 μl of culture was added to 4 ml top TY agar kept at 48^0^ C and the top agar was laid over 15 ml TY agar plate. After the agar solidified, 10 μl of SPP1 phage lysate with 10^7^/PFU was dropped on the plate. The plates were incubated at 37 C overnight. Plaque formation was measured the next day. When necessary 1 mM IPTG was added to the top agar.

### Protein expression and purification

PgcA wild type and variants were expressed from *E*. *coli* DH5α cells transformed with plasmids described in Table X and selected for by growth in LB medium containing 50 μg/mL carbenicillin. Single colonies of transformed cells were used to prepare the starting inoculum for large scale culture in 1 L of LB medium containing the same concentration of antibiotic. Cultures were incubated at 37°C with shaking until an OD_600 nm_ = 0.6 was reached. N-terminal 6×His-tagged PgcA WT or variant proteins were induced with 1 mM IPTG and expression was continued at 25°C overnight. Cells were harvested and pellets were stored at -80°C until further use. Cell pellets were suspended in binding buffer (50 mM Tris-HCl pH 7.4, 500 mM NaCl, 20 mM imidazole) and lysed using an Emulsi-Flex high pressure homogenizer at 10,000–15,000 psi. Clear lysate was separated from insoluble material by centrifugation at 10,000×g for 30 min at 4°C and loaded onto a pre-equilibrated Ni-NTA column. The column was washed with 5–7 column volumes of binding buffer prior to elution with a linear imidazole gradient and eluted at 100 mM imidazole. Relevant fractions were analyzed by SDS-PAGE to verify the purity of the protein and then pooled before buffer exchange (50 mM Tris-HCl pH 7.4, 150 mM NaCl, 10% glycerol). The protein was subsequently flash frozen in liquid nitrogen and stored at -80°C.

### Enzyme kinetic assays

An LCMS-based assay was utilized for analysis of GlmM and PgcA WT and variant activity. Prior to analysis, protein stocks were diluted in 50 mM Tris-HCl pH 8.0 containing 2 mM EDTA and allowed to pre-equilibrate for 20 mins at 30°C. Reactions (100 μL) containing 5 mM MgCl_2_, 20 μM or 0.7 mM glucose-1,6-bisphosphate for PgcA or GlmM, respectively, and 15.5 pmol (PgcA WT and G47S), 41.3 pmol GlmM or 154.6 pmol (G47D) of pure protein in 50 mM Tris-HCl pH 8.0 were initiated by the addition of glucose-1-phosphate or glucosamine-1-phosphate substrates at final concentrations ranging from 0.05–1.5 mM. At various time points (1, 5, 15 mins for GlmM; 30, 60, 90 seconds for PgcA WT and G47S; and 3, 6, 9 mins for G47D), reaction aliquots were quenched by mixing with a final concentration of 80% quenching solution (LCMS solvent B). Following centrifugation, supernatants were separated from insoluble material and 2 μL injections were subjected to LCMS analysis. All assays were monitored by the production of glucose-6-phosphate or glucosamine-6-phosphate over time and quantified by normalization to the respective standard curves.

### Mass spectrometry

LC-MS analysis was conducted using an Agilent 1290 Infinity LC system containing a Zorbax RRHD Extend C18 column (2.1 mm × 150 mm; Agilent) coupled to an Agilent Accurate Mass 6230 TOF as previously described [41]. The mobile phase consisted of solvent A (5 mM tributylamine (TBA), 5.5 mM acetic acid in 97% ddH_2_O 3% methanol) and solvent B (5 mM TBA, 5.5 mM acetic acid in methanol) run at a flow rate of 0.25 mL/min with the following gradient: 0–3.5 min, 0% B; 4–7.5 min, 30% B; 7.5–8 min, 35% B; and 12–16 min, 99% B; followed by a 5 min equilibration period at 0% solvent B prior to injection of the next sample. Dynamic mass axis calibration was accomplished by continuous infusion of a reference mass solution. ESI capillary and fragmentor voltages were set at 4000 V and 125 V respectively. The nebulizer pressure was set to 45 psig and nitrogen drying gas was set to a flow rate of 8 L/min. The drying gas temperature was maintained at 325°C. The MS acquisition rate was 1.5 spectra/ sec and m/z data ranging from 50–1100 was stored. Data was analyzed using Profinder B.08.00 software, and ions were assigned as specific metabolites based on mass accuracy within 5 parts per million (ppm) and retention times within 1 min of those determined for chemical standards.

## Supporting information

S1 Fig Growth of WT, Δ*glmR*, Δ*gtaB* and Δ*glmR* Δ*gtaB* on LB agar with 20 mM MgSO_4_. (TIFF)

S2 Fig **(A)** Enzymatic activity of purified PgcA variants (WT, G47S, G47D) and GlmM as measured in the PGM reaction using glucose 1-phosphate (varied from 0.05 to 1.0 mM) as substrate. **(B)** PNGM activity of PgcA variants and GlmM using GlcN-1-P as substrate (varied from 0.1 to 1.5 mM). The right panel is an enlarged version of the left panel to allow visualization of the low activity of GlmM. The error bars represent standard deviation of the average value of three biological replicates. **(C)** Summary S1 Table of data used for panels (A) and (B) with average specific PGM and PNGM activity from three biological replicates and their standard deviation.
(TIFF)

S3 Fig Summary of kinetic parameters (K_M_, k_cat_ and catalytic efficiency) for PgcA variants and GlmM in (**A**) PGM reaction and (**B**) PNGM reaction. (C) Summary S2 Table of average value of K_M_, k_cat_ and catalytic efficiency and standard deviation from three biological replicates.
(TIFF)

S4 Fig Sequence alignment of phosphoglucomutases from different bacteria. Glycine and alanine corresponding to G47 in *B*. *subtilis* PgcA and related proteins (the PGM_Bs_ group) is highlighted in yellow, and corresponding serine present in *E*. *coli* Pgm and related proteins (the PGM_Ec_ group) is highlighted in blue.
(TIFF)

S5 Fig **(A)** Sequence alignment of *B*. *subtilis* PgcA and GlmM. The residues highlighted in green indicate G47 of PgcA and aspartate (D) at the same position in GlmM, **(B)** Sequence alignment of phosphoglucosamine mutase (GlmM) from different bacteria. The analysis was done with Clustal omega.
(TIFF)

S6 Fig **(A)** Images of LB agar plates with 20 mM MgSO_4_ plates where WT, *pgcA*_G47S_ and *pgcA*_G47D_ were transformed with *glmR*::*mls*. These representative images were taken after overnight incubation at 37^0^ C. **(B)** CEF sensitivity assay was carried out with 6 μg of antibiotic. 1 mM IPTG was added for induction of *pgcA*_G47D_. **(C)** Images showing SPP1 phage infection assay. Cell lysis was observed after overnight incubation at 37^0^ C. 10 μl of 10^7^ PFU/ml SPP1 was spotted on the lawns *B*. *subtilis* strains of interest. Shown pictures are representative of at least three biological replicates.
(TIFF)

S7 Fig Growth assay on gluconeogenic MH medium carried out with and without 1 mM IPTG. Pictures were taken after overnight incubation of plates at 37^0^ C. These images are representative of at least three biological replicates.
(TIFF)

S8 Fig Multiple sequence alignment carried out using Clustal Omega for *Trypanosoma brucei* phosphoglucosamine mutase with *B*. *subtilis* PgcA and GlmM. Residues corresponding to *B*. *subtilis* G47 are highlighted in blue. (TIFF)

S1 Table Summary of data displayed in S2 Fig. (TIFF)

S2 Table Summary of data displayed in S3 Fig. (TIFF)

S3 Table Primers used in this study. (TIFF)
