# Supplementary figures and images for: Bacillus subtilis PgcA moonlights as a phosphoglucosamine mutase in support of peptidoglycan synthesis
Source: PLoS Genet. 2019 Oct 7;15(10):e1008434. doi: 10.1371/journal.pgen.1008434 (PMC6797236; doi:10.1371/journal.pgen.1008434)

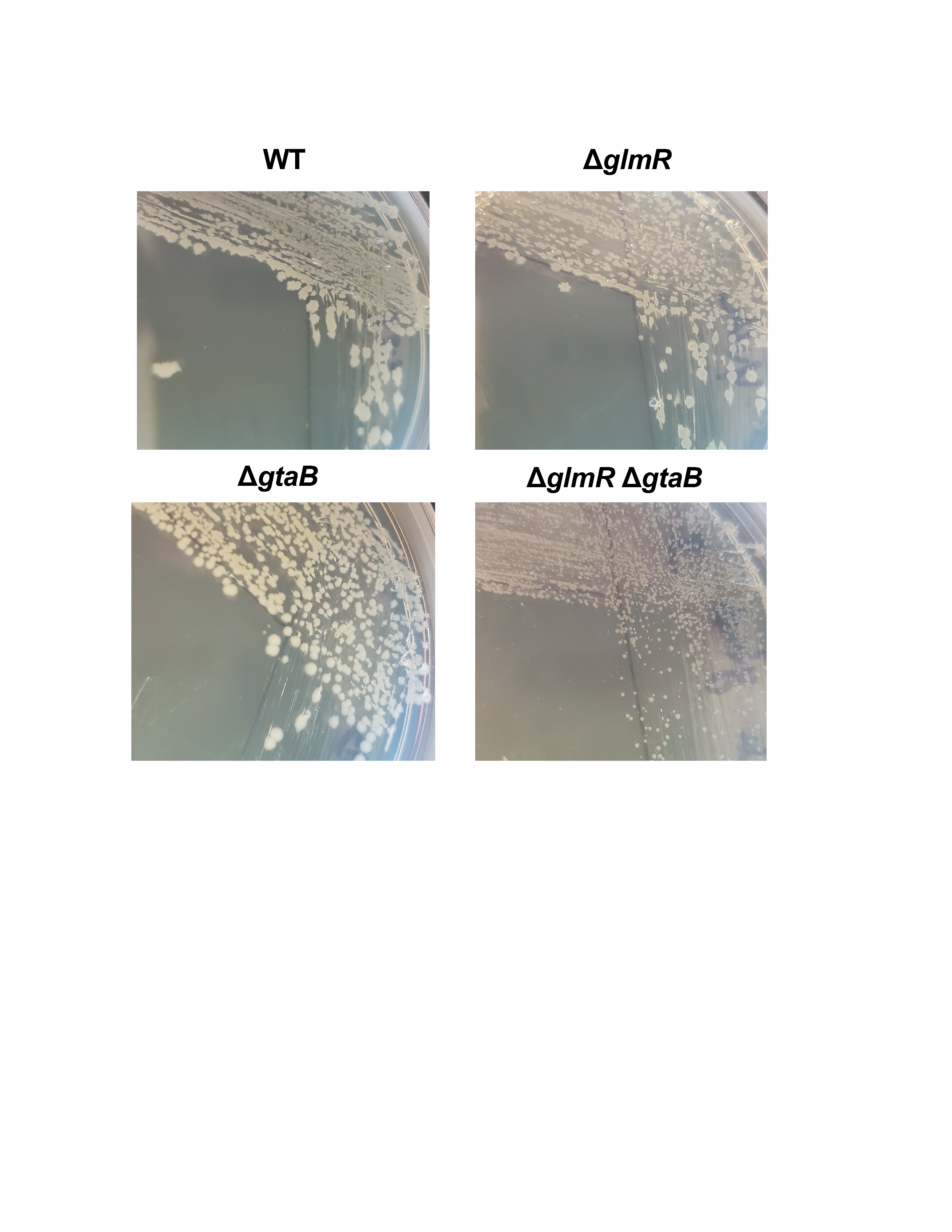

Supplement: S1 Fig — (TIFF) [file pgen.1008434.s001.tiff]

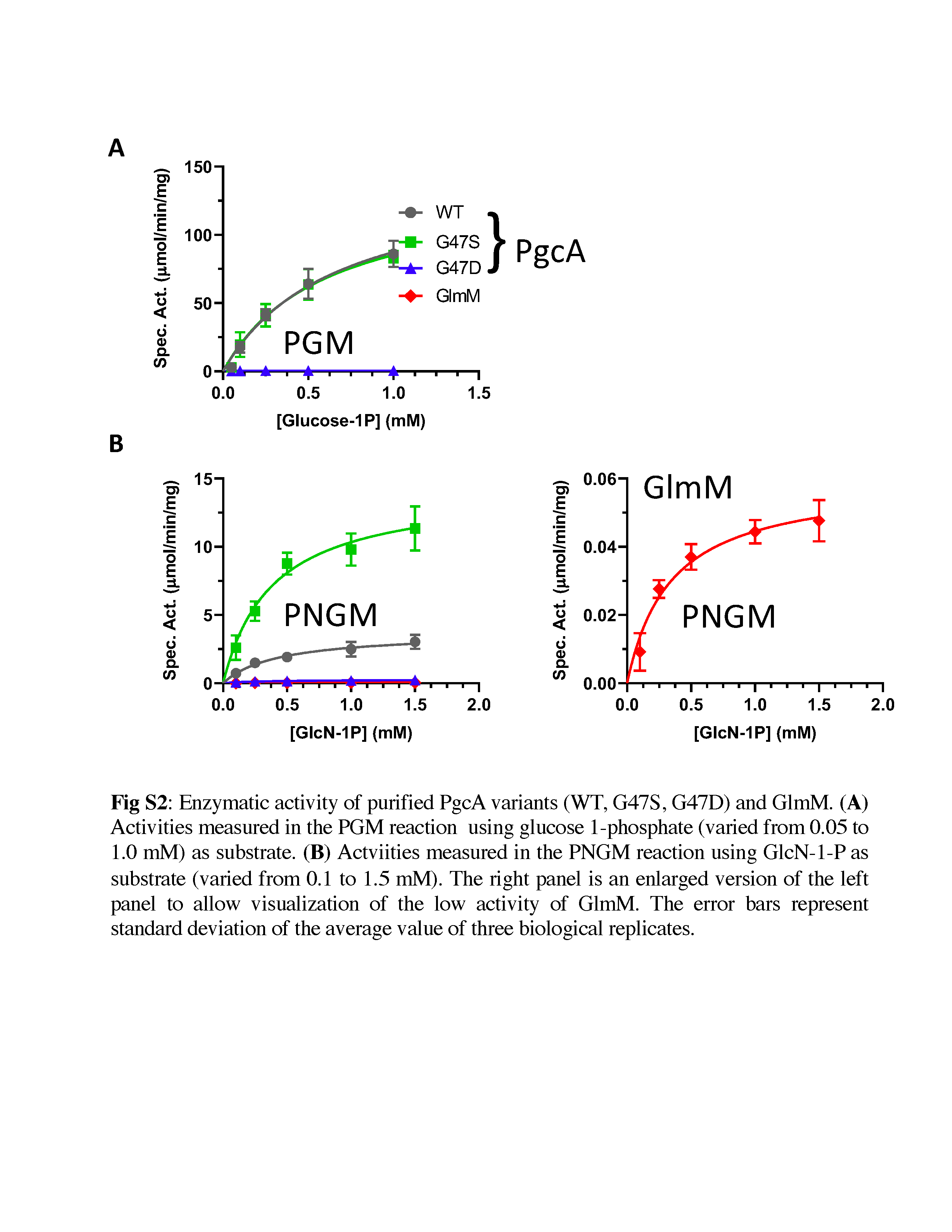

Supplement: S2 Fig — (A) Enzymatic activity of purified PgcA variants (WT, G47S, G47D) and GlmM as measured in the PGM reaction using glucose 1-phosphate (varied from 0.05 to 1.0 mM) as substrate. (B) PNGM activity of PgcA variants and GlmM using GlcN-1-P as substrate (varied from 0.1 to 1.5 mM). The right panel is an enlarged version of the left panel to allow visualization of the low activity of GlmM. The error bars represent standard deviation of the average value of three biological replicates. (C) Summary S1 Table of data used for panels (A) and (B) with average specific PGM and PNGM activity from three biological replicates and their standard deviation. (TIFF) [file pgen.1008434.s002.tiff]

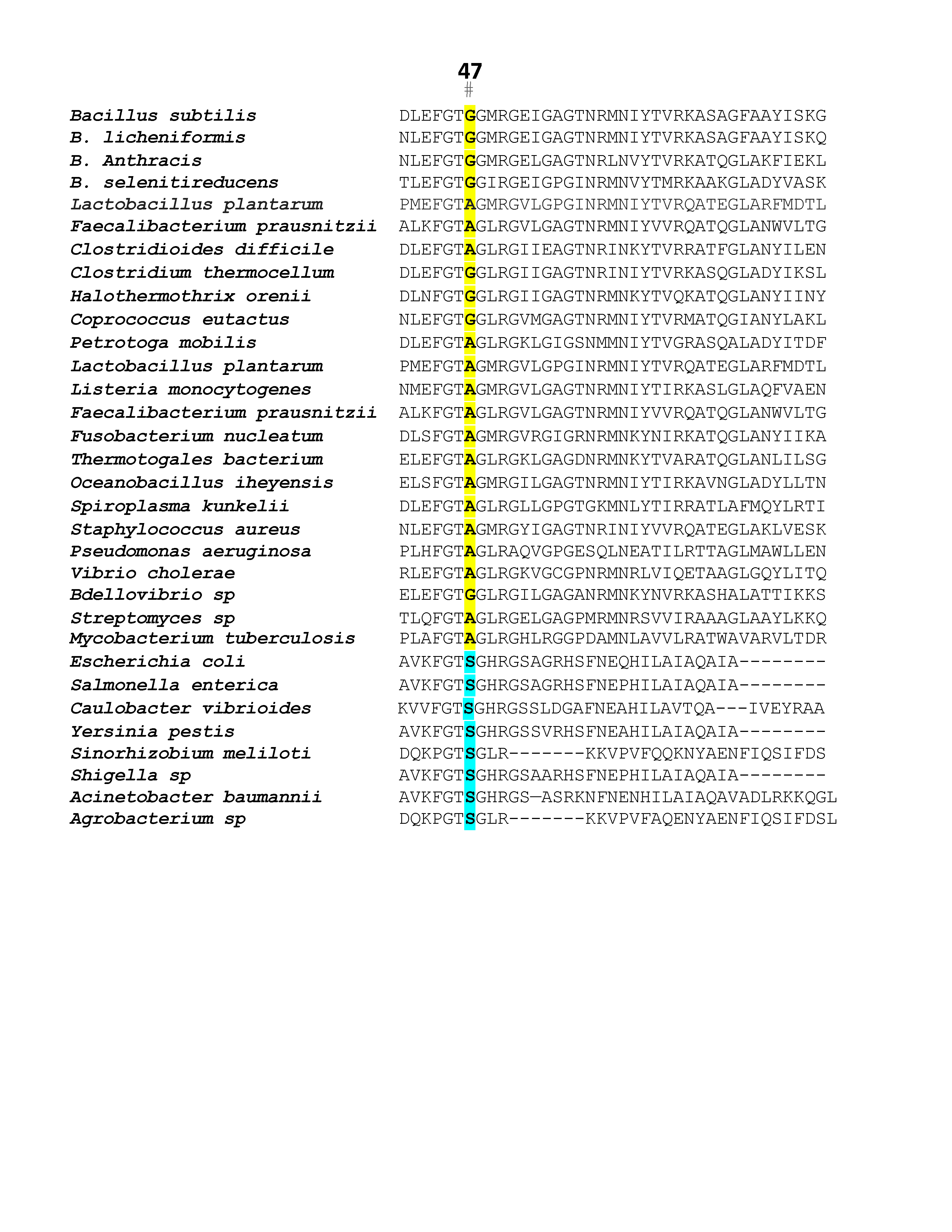

Supplement: S4 Fig — Glycine and alanine corresponding to G47 in B. subtilis PgcA and related proteins (the PGMBs group) is highlighted in yellow, and corresponding serine present in E. coli Pgm and related proteins (the PGMEc group) is highlighted in blue. (TIFF) [file pgen.1008434.s004.tiff]

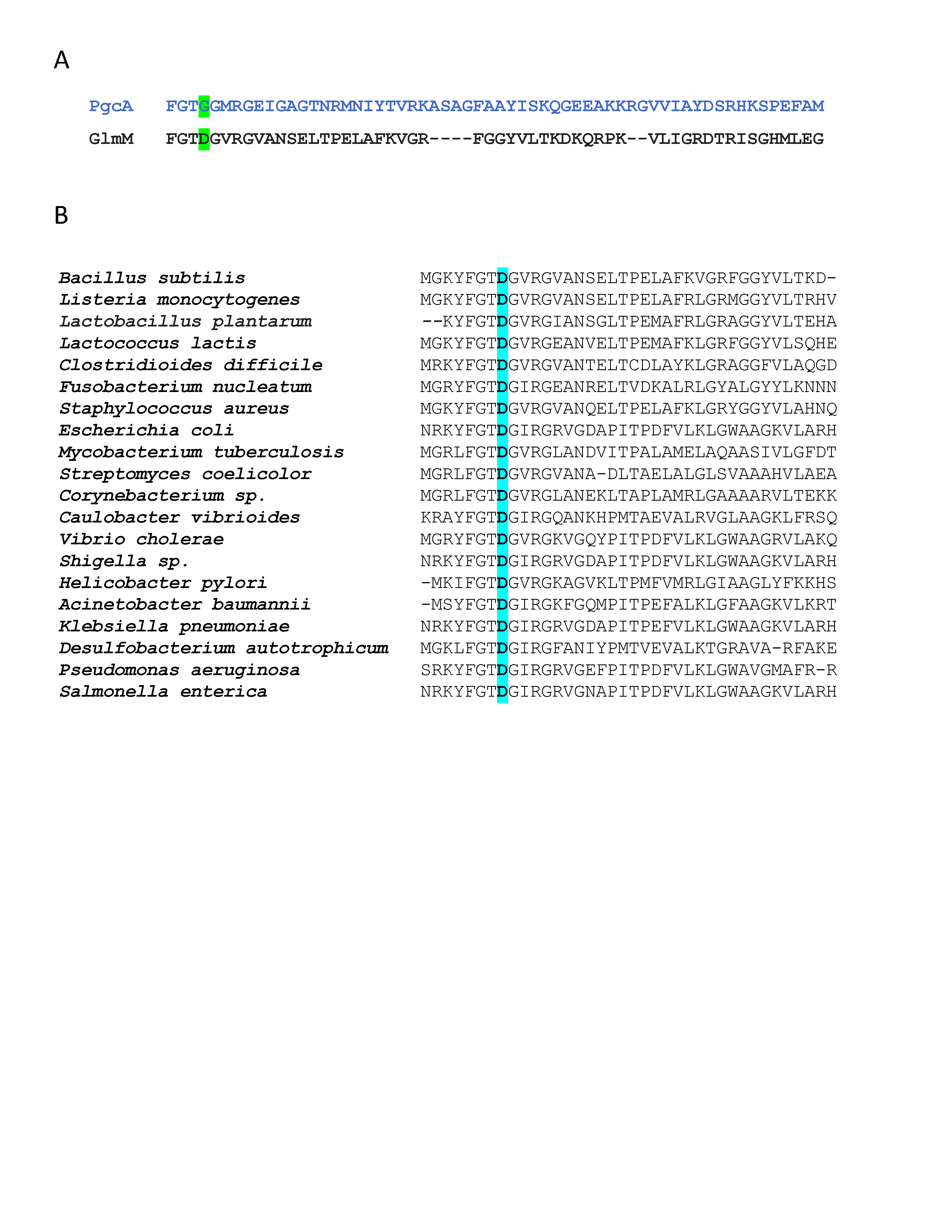

Supplement: S5 Fig — (A) Sequence alignment of B. subtilis PgcA and GlmM. The residues highlighted in green indicate G47 of PgcA and aspartate (D) at the same position in GlmM, (B) Sequence alignment of phosphoglucosamine mutase (GlmM) from different bacteria. The analysis was done with Clustal omega. (TIFF) [file pgen.1008434.s005.tiff]

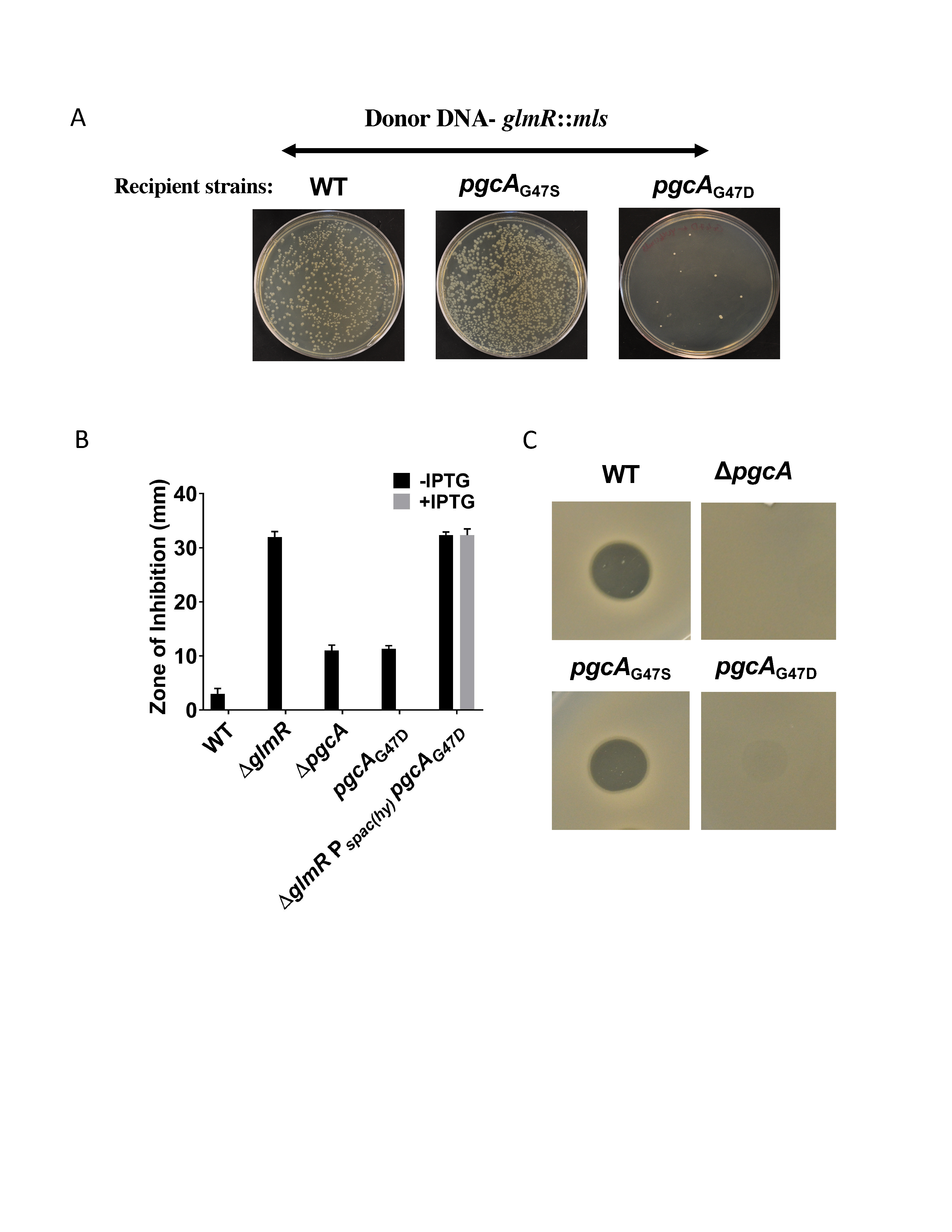

Supplement: S6 Fig — (A) Images of LB agar plates with 20 mM MgSO4 plates where WT, pgcAG47S and pgcAG47D were transformed with glmR::mls. These representative images were taken after overnight incubation at 370 C. (B) CEF sensitivity assay was carried out with 6 μg of antibiotic. 1 mM IPTG was added for induction of pgcAG47D. (C) Images showing SPP1 phage infection assay. Cell lysis was observed after overnight incubation at 370 C. 10 μl of 107 PFU/ml SPP1 was spotted on the lawns B. subtilis strains of interest. Shown pictures are representative of at least three biological replicates. (TIFF) [file pgen.1008434.s006.tiff]

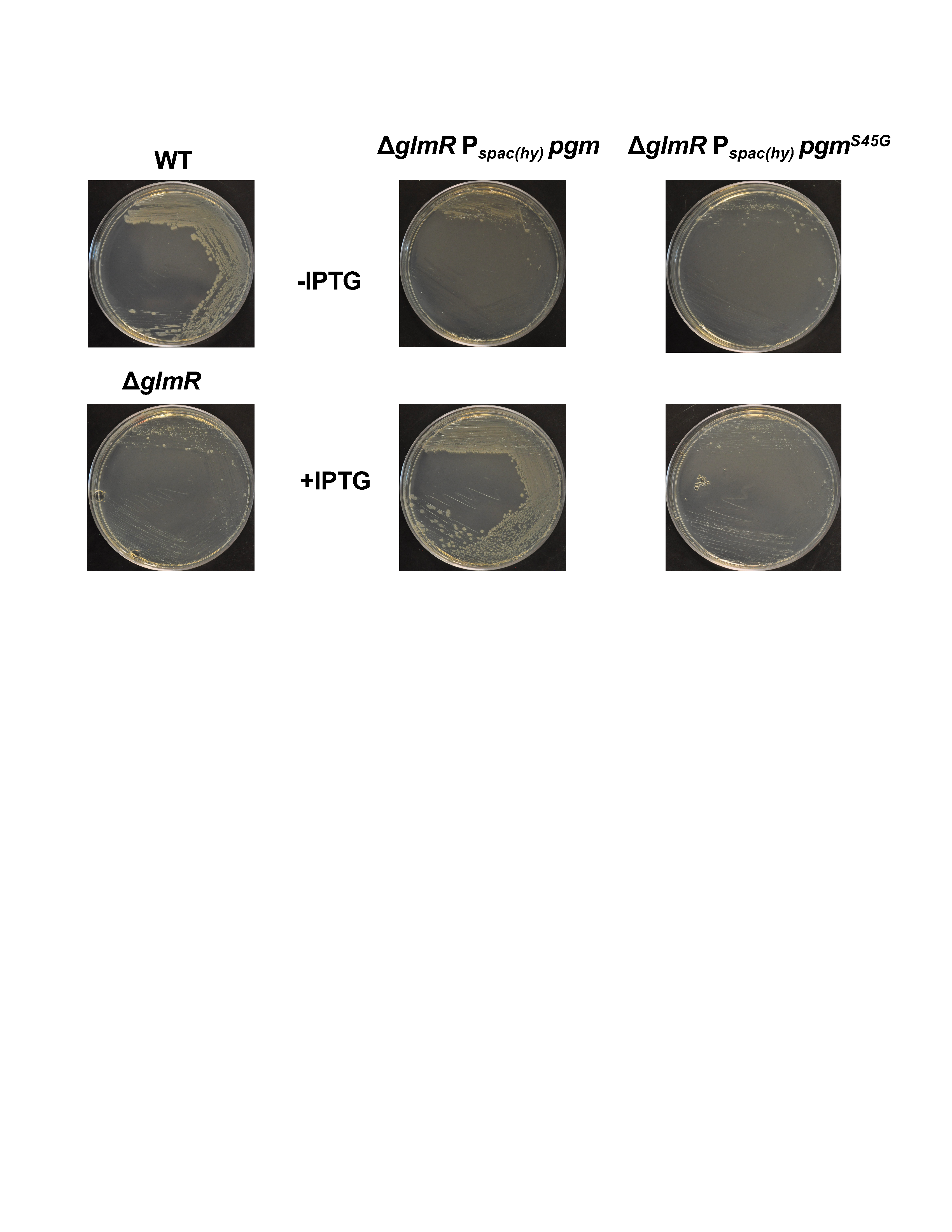

Supplement: S7 Fig — Pictures were taken after overnight incubation of plates at 370 C. These images are representative of at least three biological replicates. (TIFF) [file pgen.1008434.s007.tiff]

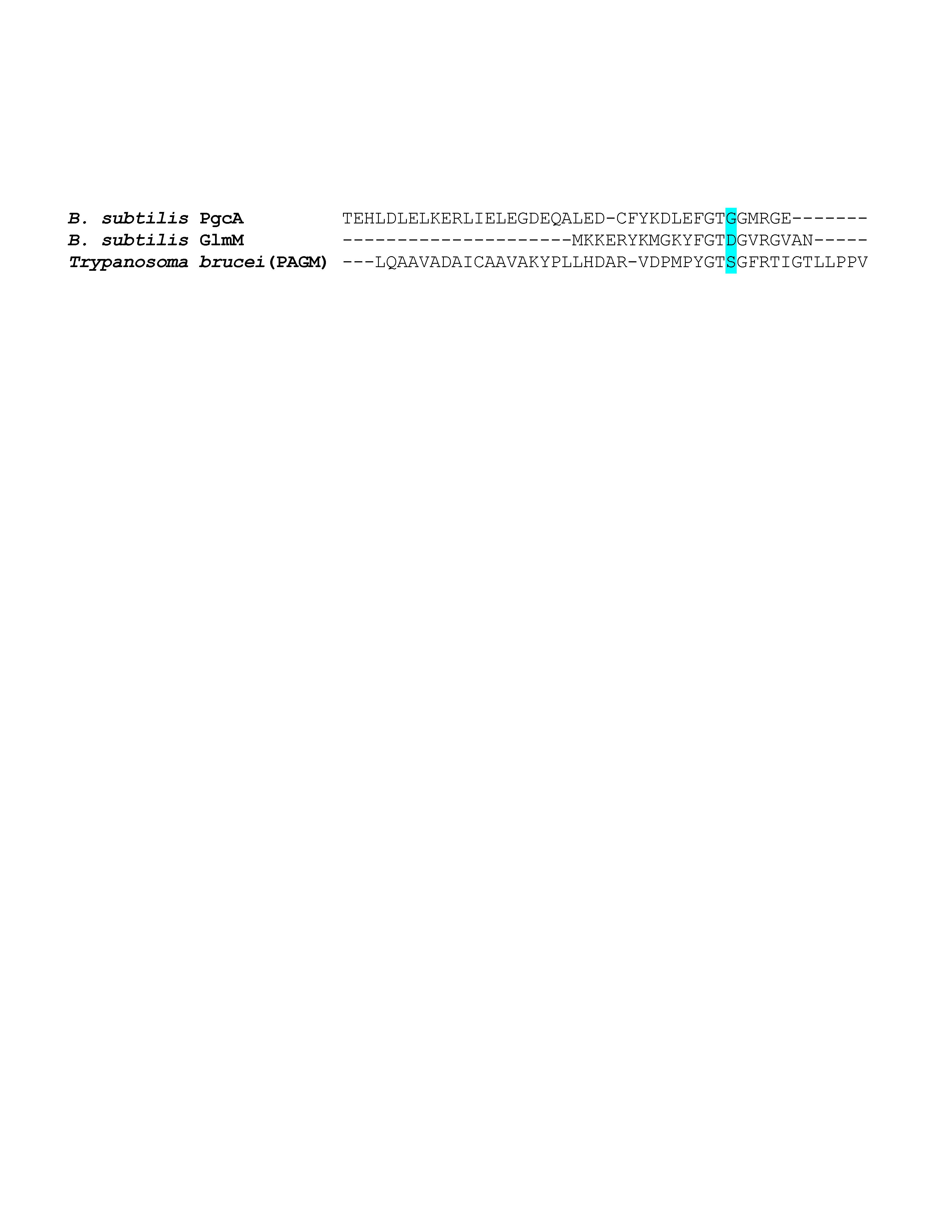

Supplement: S8 Fig — (TIFF) [file pgen.1008434.s008.tiff]

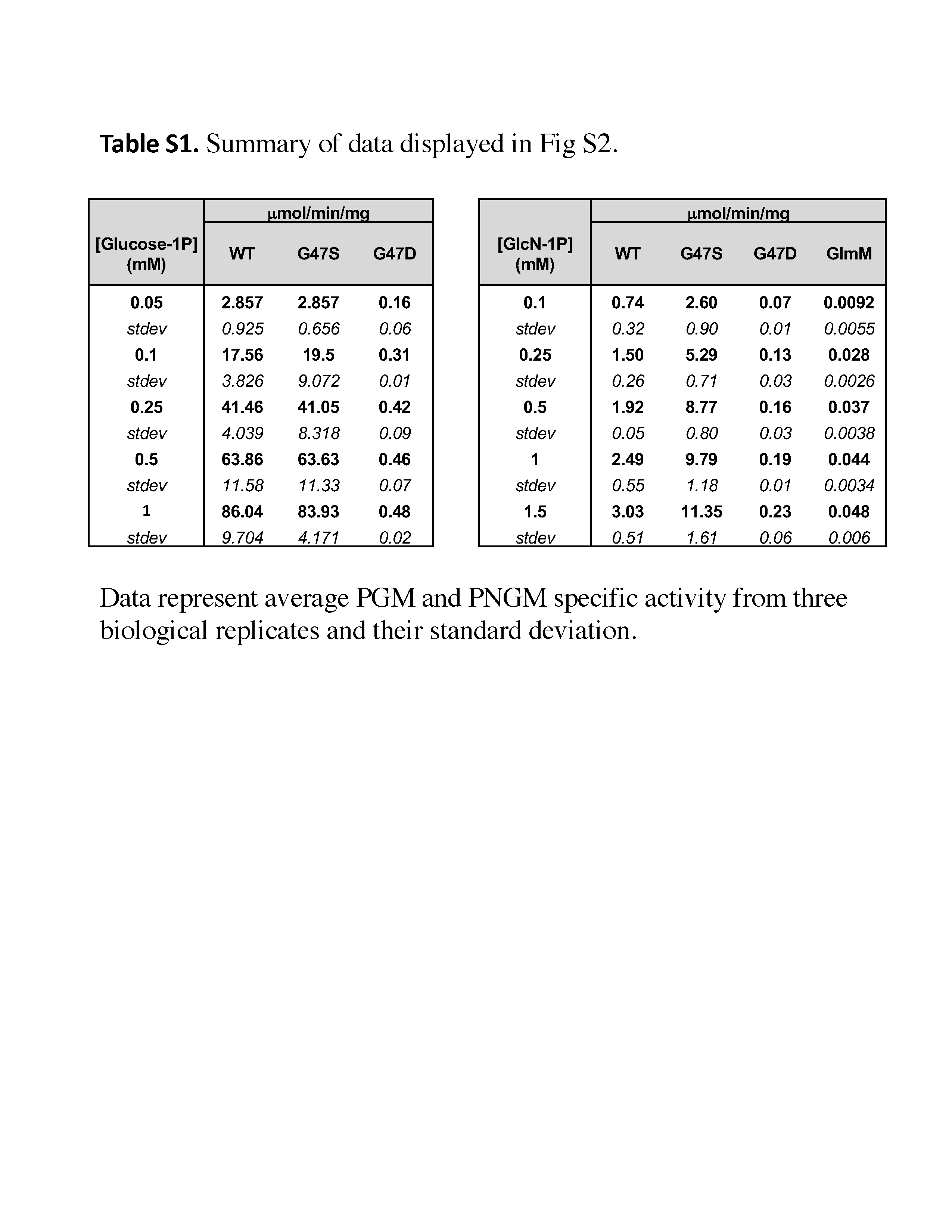

Supplement: S1 Table — (TIFF) [file pgen.1008434.s009.tiff]

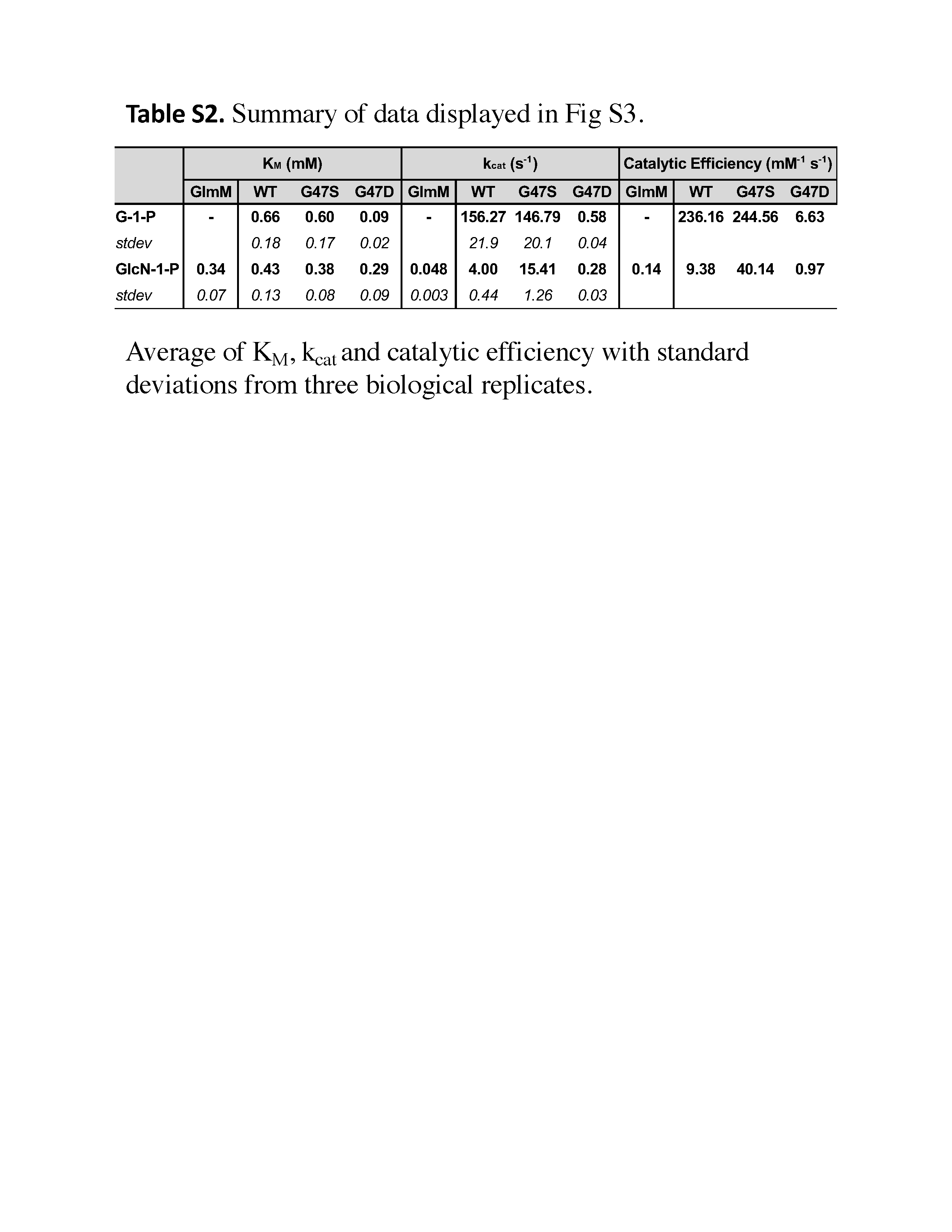

Supplement: S2 Table — (TIFF) [file pgen.1008434.s010.tiff]
